# Supplementary material for: PP2A phosphatase regulates cell-type specific cytoskeletal organization to drive dendrite diversity
Source: Front Mol Neurosci. 2022 Nov 14;15:926567. doi: 10.3389/fnmol.2022.926567 (PMC9702092; doi:10.3389/fnmol.2022.926567)
Supplement: Supplementary file 1 [file Data_Sheet_1.PDF]

Supplementary Material

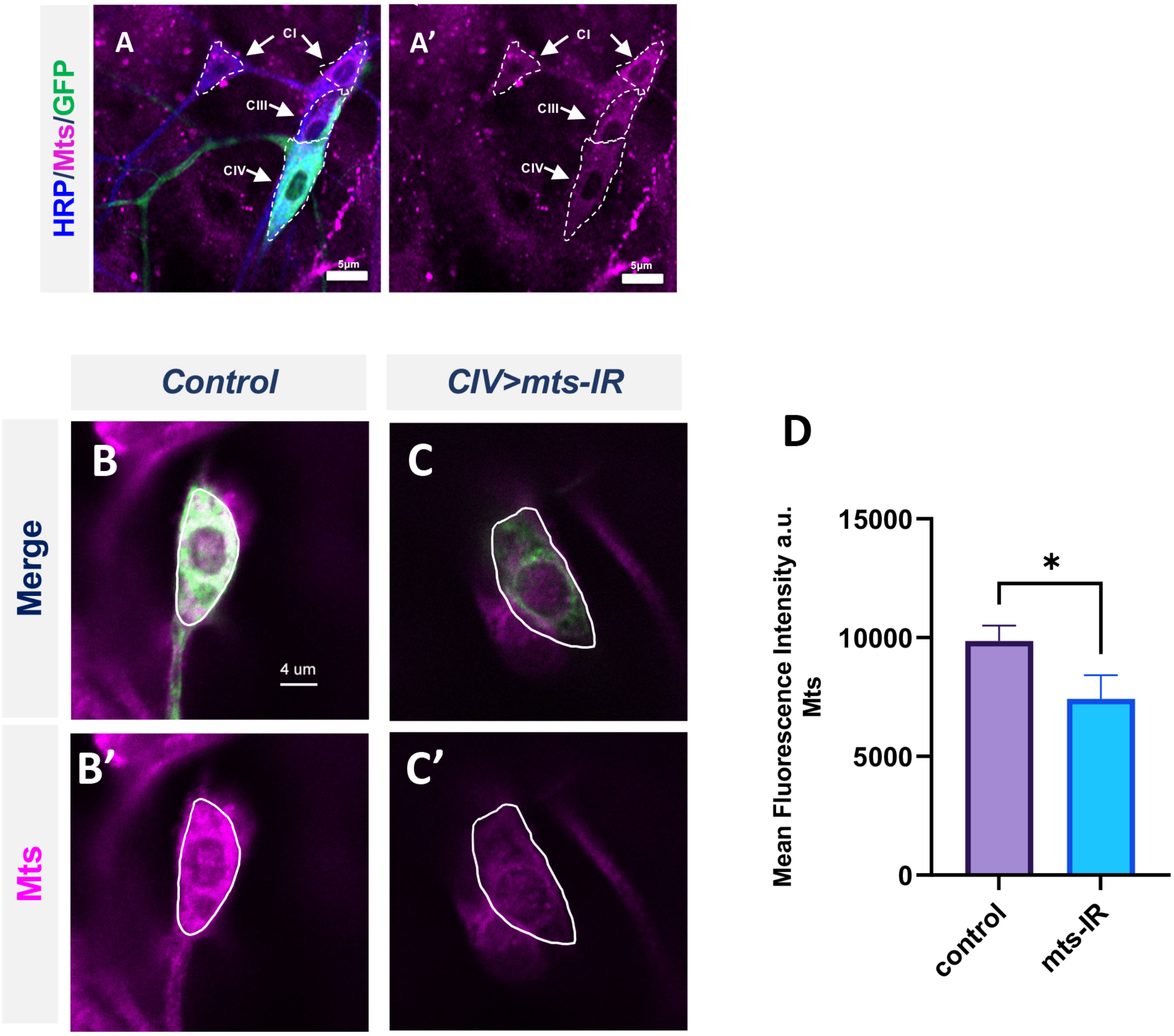

Supplementary Figure 1: ***PP2A is expressed in md neurons*: (A, A')** Immunohistochemical analysis shows that Mts is expressed in md neurons. Representative images of control **(B, B')** and *mts-IR* CIV neurons **(C, C')**. **(D)** Quantittive immunohistochemical analysis of Mts expression in control and *mts-IR*. Sholl quantitative morphometric analyses. Statistical tests performed: **(D)** unpaired t-test (n = 18, 5) *=p≤0.05. For detailed genotypes see Supplementary Table 1 and for detailed statistics see Supplementary Table 2. Scale bar = 5 µm for **(A-A')**, 3 µm for **(B)**.

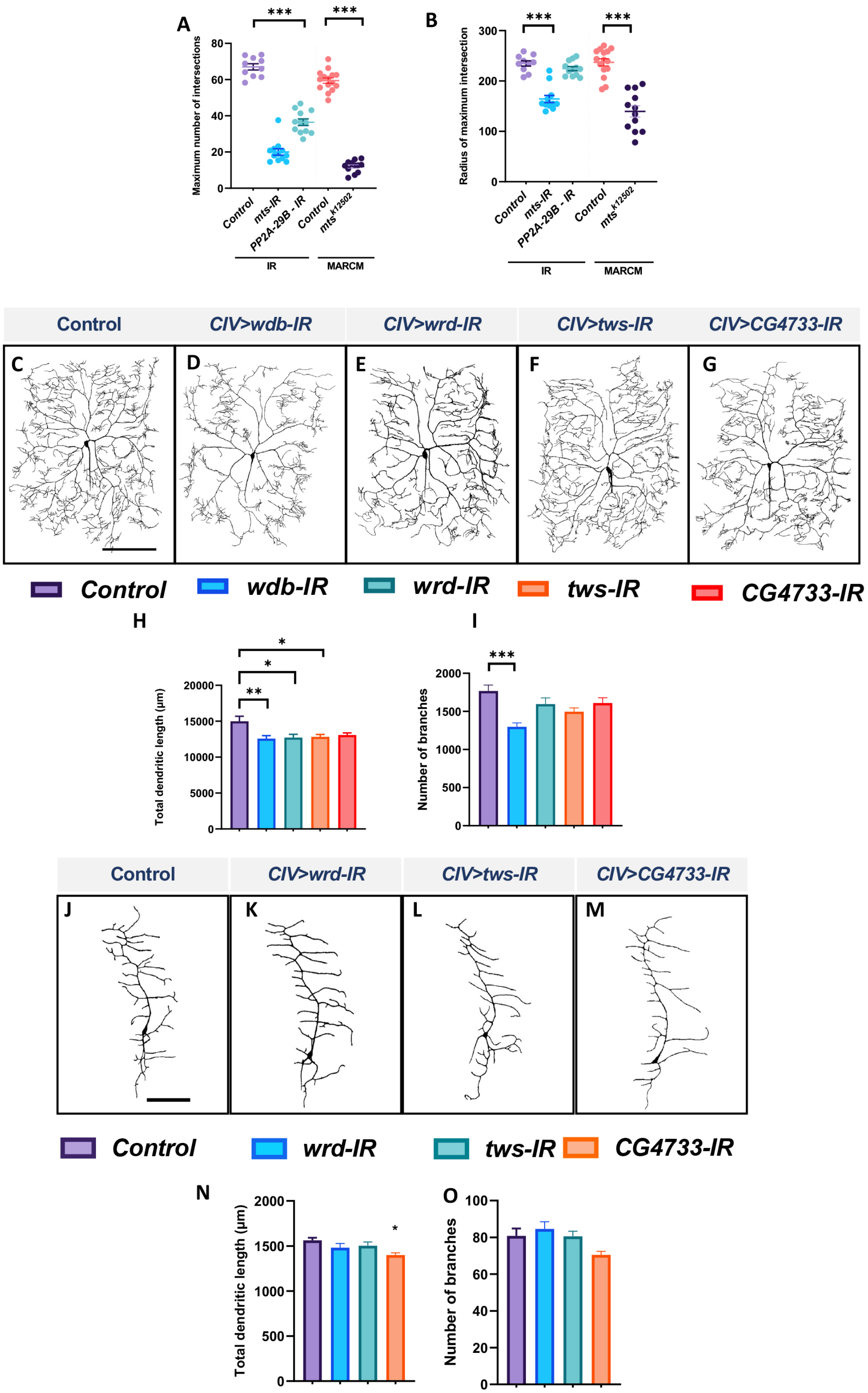
A
***
***
Maximum number of intersections
80
60
40
20
0
Control
mts-IR
PP2A-29B - IR
Control
mts k12502
IR
MARCM
B
***
***
Radius of maximum intersection
300
200
100
0
Control
mts-IR
PP2A-29B - IR
Control
mts k12502
IR
MARCM
Control
CIV>wdb-IR
CIV>wrd-IR
CIV>tws-IR
CIV>CG4733-IR
C
D
E
F
G
Control
wdb-IR
wrd-IR
tws-IR
CG4733-IR
H
*
*
**
Total dendritic length (µm)
20000
15000
10000
5000
0
I
***
Number of branches
2000
1500
1000
500
0
Control
CIV>wrd-IR
CIV>tws-IR
CIV>CG4733-IR
J
K
L
M
Control
wrd-IR
tws-IR
CG4733-IR
N
Total dendritic length (µm)
2000
1500
1000
500
0
*
O
Number of branches
100
80
60
40
20
0

Supplementary Figure 2: ***Regulatory subunits of PP2A have mild effects on dendritic morphology*****:** **(A, B)** Sholl quantitative morphometric analyses. Representative images of CIV neurons in **(C)** control, **(D)** *wdb-IR* **(E)** *wrd-IR*, **(F)** *tws-IR* **(G)** *CG4733-IR*. **(H, I)** Quantitative morphometric analyses. Representative images of CI neurons in **(J)** control, **(K)** *wrd-IR*, **(L)** *tws-IR* **(M)** *CG4733-IR*. **(N, O)** Quantitative morphometric analyses. Statistical tests performed: **(A, B, I, N, O)** Kruskal-Wallis with Dunn's multiple comparisons test (n = 10-16 per genotype), **(H)** One-way ANOVA with Sidak's multiple comparisons test (n = 10-16 per genotype). ***=$p \leq 0.001$, **=$p \leq 0.01$, *=$p \leq 0.05$. For detailed genotypes see Supplementary Table 1 and for detailed statistics see Supplementary Table 2. Scale bar = 200 μm for **(C)**, and 100 μm for **(J).**

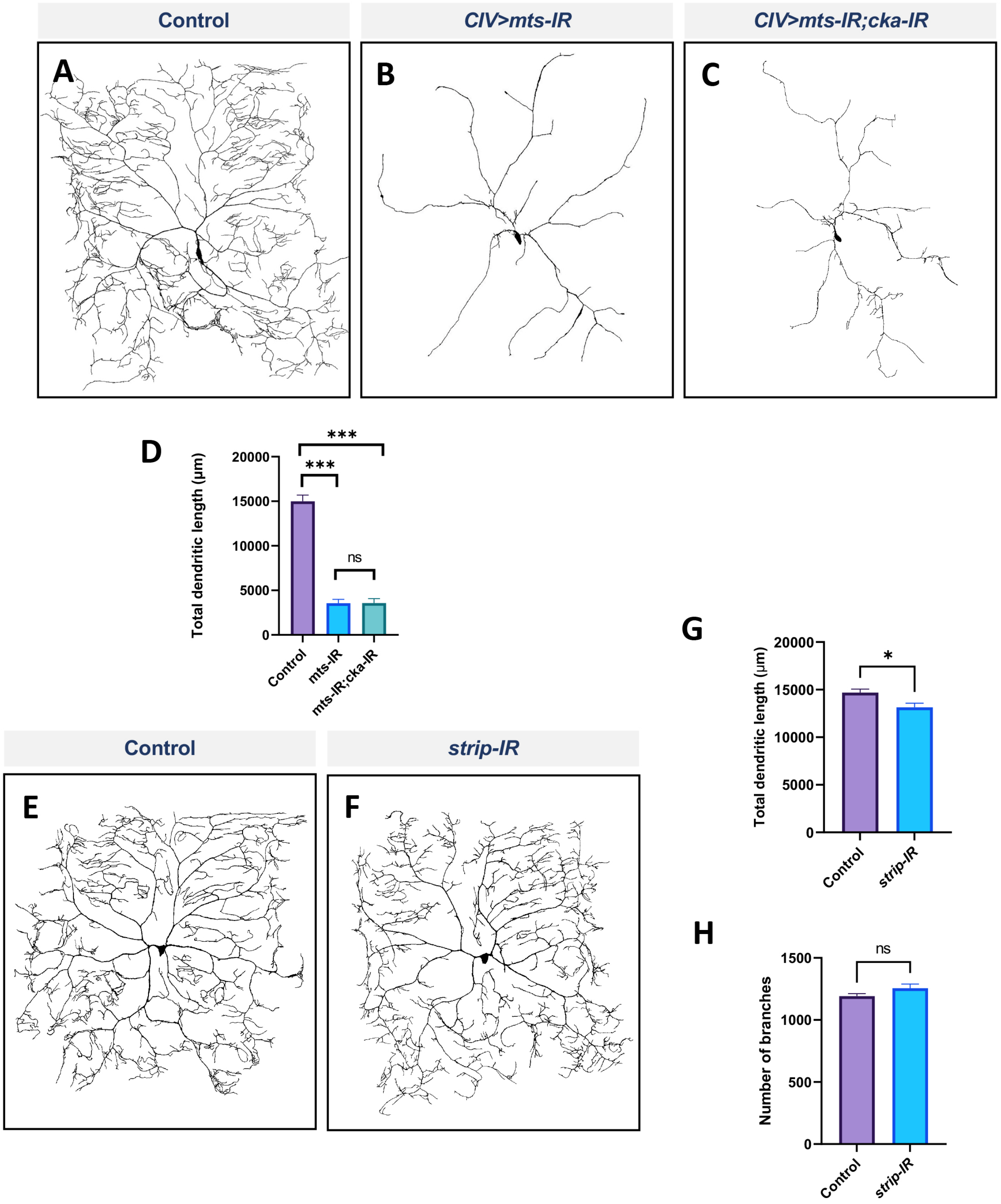

Control
CIV>mts-IR
CIV>mts-IR;cka-IR
A
B
C
D
Total dendritic length (µm)
20000
15000
10000
5000
0
***
***
ns
Control
mts-IR
mts-IR;cka-IR
Control
strip-IR
E
F
G
Total dendritic length (µm)
20000
15000
10000
5000
0
*
Control
strip-IR
H
Number of branches
1500
1000
500
0
ns
Control
strip-IR

Supplementary Figure 3: ***PP2A and STRIPAK function in parallel to regulate morphology:*** Representative images of CIV neurons in **(A)** control, **(B)** *mts-IR* **(C)** *mts-IR;cka-IR*. **(D)** Quantitative morphometric analysis. Representative images of CI neurons in **(E)** control, **(F)** *strip-IR*. **(G, H)** Quantitative morphometric analyses. Statistical tests performed: **(D)** One-way ANOVA with Sidak's multiple comparisons test (n = 9-11 per genotype). **(G, H)** Unpaired t-test (n=9, 18) ***=p≤0.001, *=p≤0.05. For detailed genotypes see Supplementary Table 1 and for detailed statistics see Supplementary Table 2. Scale bar = 200 µm for **(A, E).**

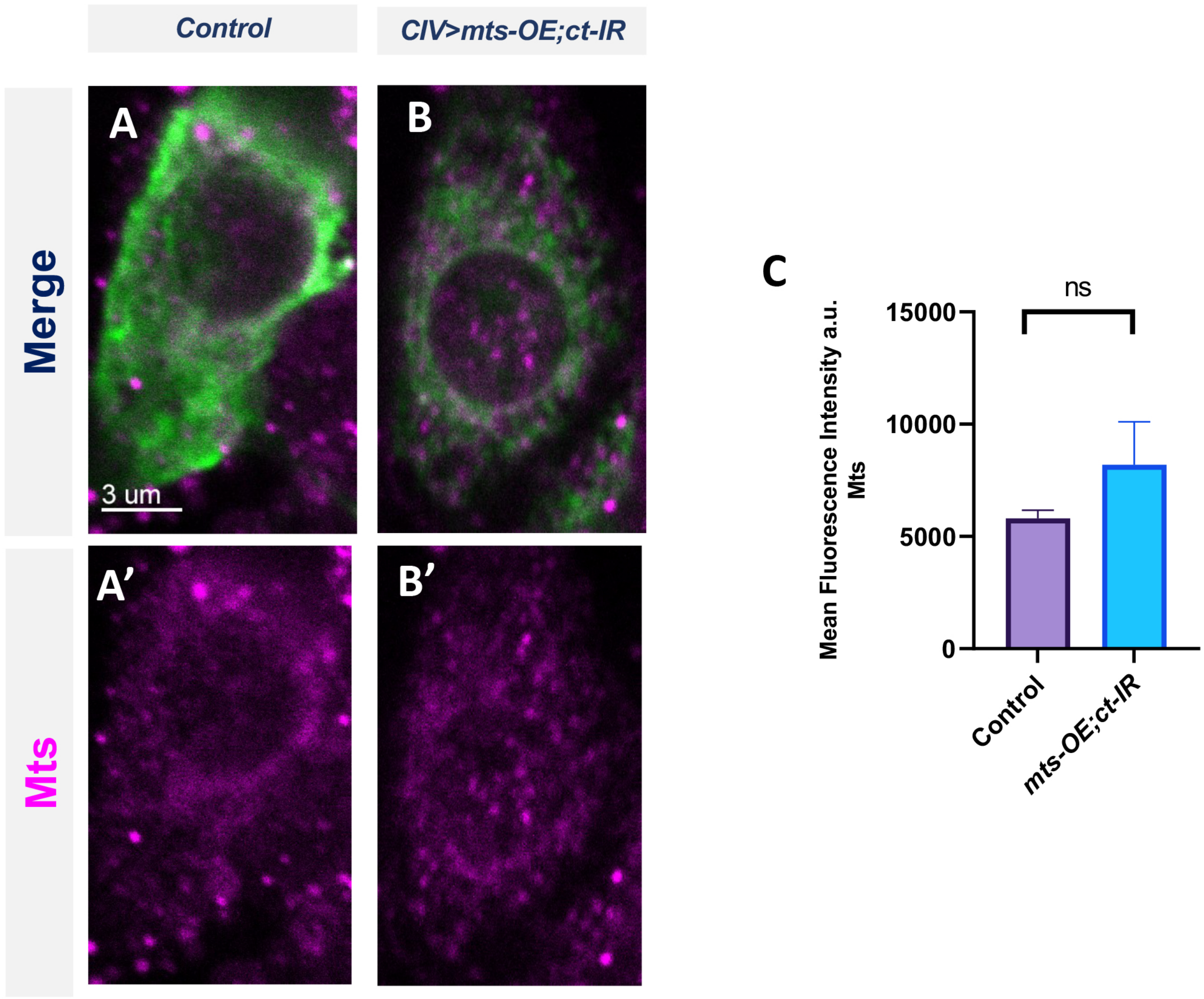

Supplementary Figure 4: ***Mts expression in mts-OE;ct-IR is not significantly different from control :*** Representative images of CIV neurons in **(A, A')** control, **(B, B')** *mts-OE;ct-IR* **((C)** Quantitative IHC analysis. Statistical tests performed: **(C)** Unpaired t-test (n=13,8). For detailed genotypes see Supplementary Table 1 and for detailed statistics see Supplementary Table 2. Scale bar = 3µm for **(A).**

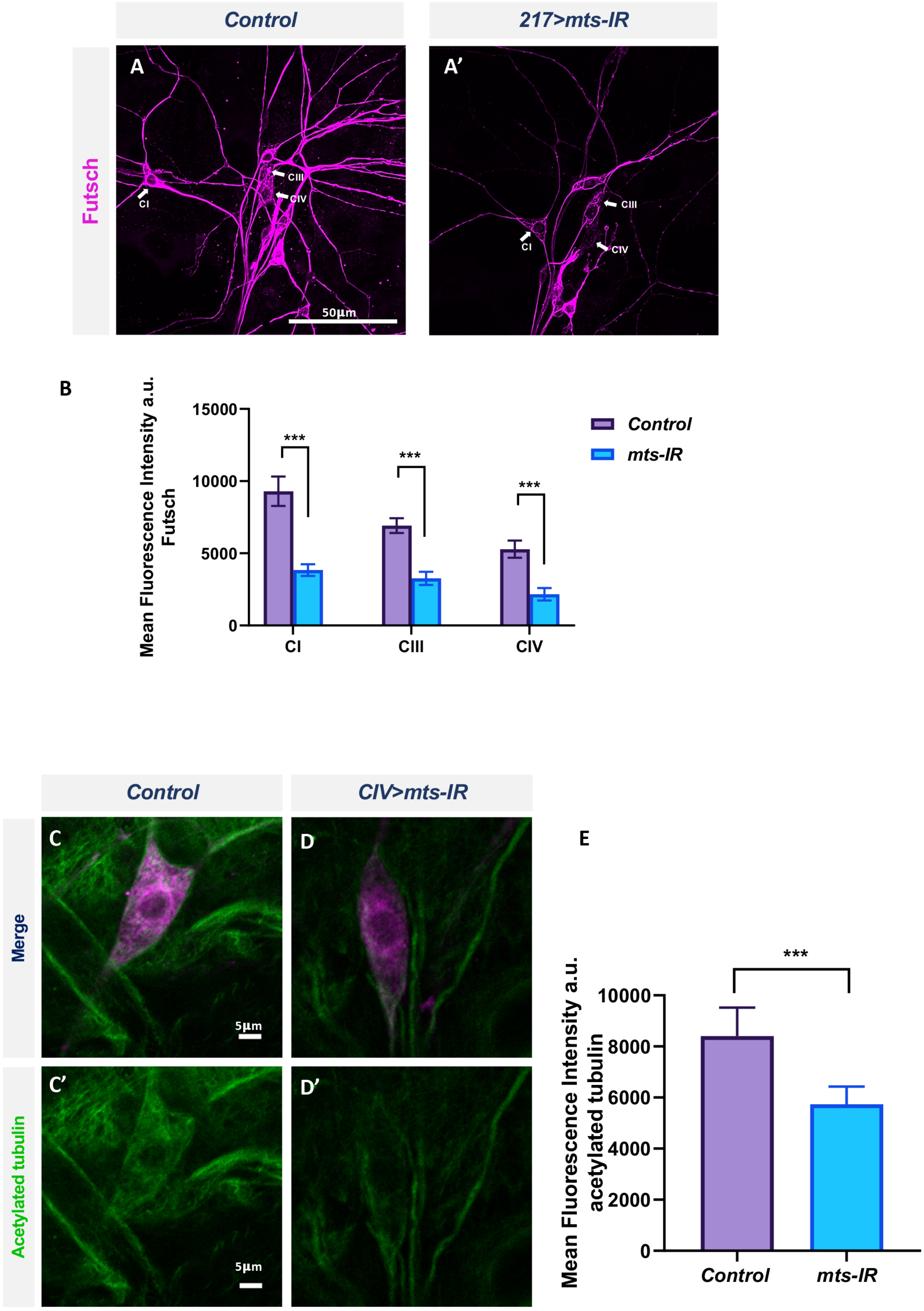
Control
217>mts-IR
Futsch
A
A'
CI
CIII
CIV
50µm
B
Mean Fluorescence Intensity a.u.
Futsch
15000
10000
5000
0
***
CI
CIII
CIV
Control
mts-IR
Control
CIV>mts-IR
Merge
Acetylated tubulin
C
C'
D
D'
5µm
E
Mean Fluorescence Intensity a.u.
acetylated tubulin
10000
8000
6000
4000
2000
0
***
Control
mts-IR

Supplementary Figure 5: ***PP2A is required for MT stabilization:*** Representative images of immunohistochemical analyses showing the expression of Futsch in md neurons in **(A)** control and **(A')** *mts-IR* animals. **(B)** Quantitative analysis of the mean fluorescence intensities of Futsch signaling normalized to area in md neurons. Representative images of immunohistochemical analysis showing the expression of acetylated tubulin in CIV neurons in **(C, C')** control and **(D, D')** *mts-IR* animals. **(E)** Quantitative analysis of the mean fluorescence intensities of acetylated tubulin normalized to area. Statistical tests performed: **(B)** Two-way ANOVA with Dunnett's multiple comparisons test (n = 14-18 per genotype), **(E)** Student's t-test (n = 11-16 per genotype). ***=$p \leq 0.001$. For detailed genotypes see Supplementary Table 1 and for detailed statistics see Supplementary Table 2. Scale bar = 50 µm for **(A)** and 5 µm for **(C-C')**.

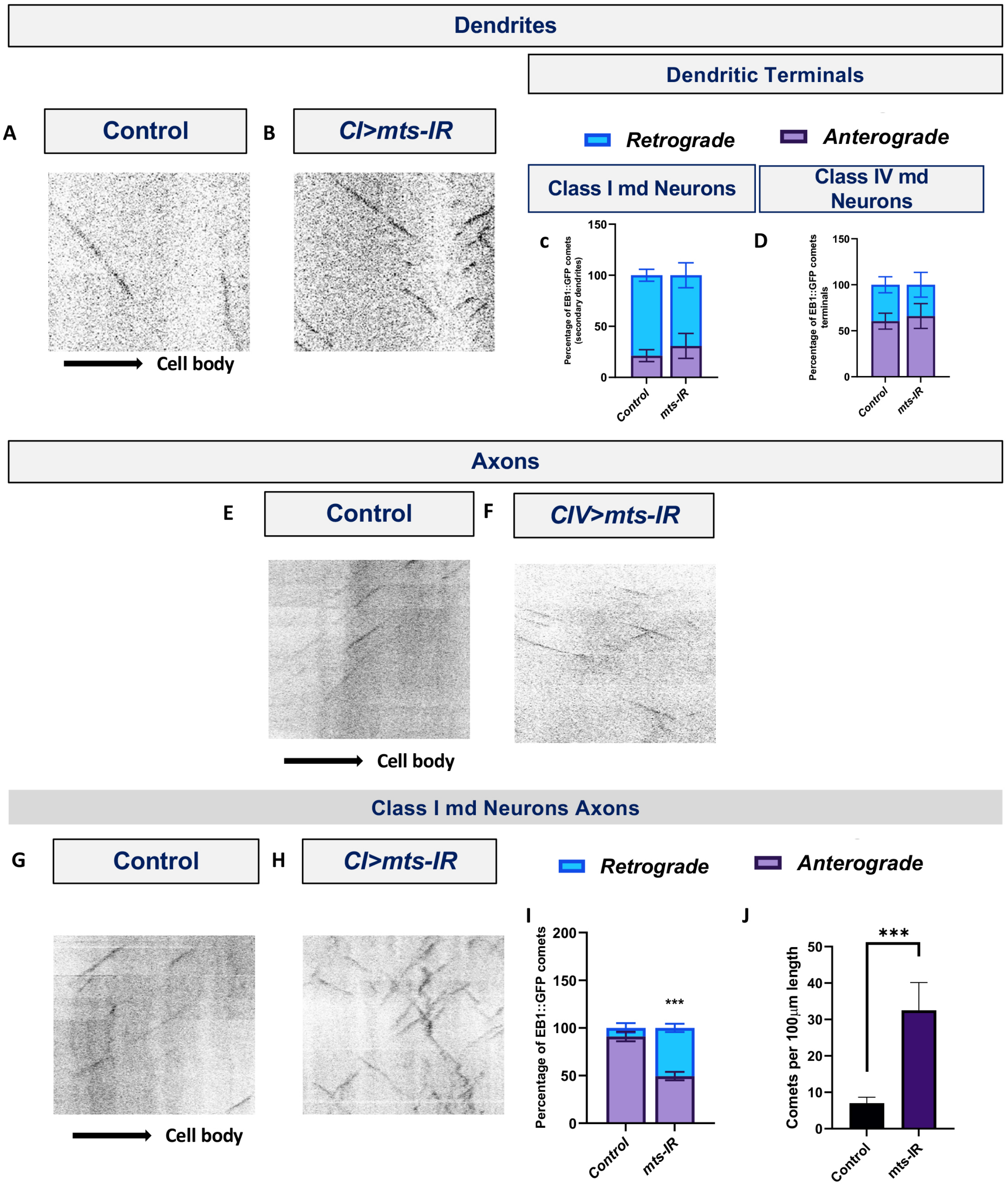

Dendrites
Dendritic Terminals
A
Control
B
CI>mts-IR
Cell body
Retrograde
Anterograde
Class I md Neurons
Class IV md Neurons
C
Percentage of EB1::GFP comets (secondary dendrites)
150
100
50
0
Control
mts-IR
D
Percentage of EB1::GFP comets terminals
150
100
50
0
Control
mts-IR
Axons
E
Control
F
CIV>mts-IR
Cell body
Class I md Neurons Axons
G
Control
H
CI>mts-IR
Cell body
Retrograde
Anterograde
I
Percentage of EB1::GFP comets
200
150
100
50
0
***
Control
mts-IR
J
Comets per 100μm length
50
40
30
20
10
0
***
Control
mts-IR

Supplementary Figure 6: ***PP2A knockdown disrupts MT polarity in both axons and dendrites of md neurons but not dendritic terminals:*** Kymographs showing the EB1::GFP comet trajectories in dendrites of CI neurons in **(A)** control and **(B)** *mts-IR*. **(C, D)** Knockdown of *mts* in CI or CIV md neurons does not affect MT polarity at dendritic terminals. Representative kymographs of EB1::GFP comets in axons of CIV and CI neurons in **(E, G)** control and **(F, H)** *mts-IR* respectively. **(I, J)** Compared to controls, knockdown of *mts* reverses MT polarity in CI axons along with an increase in the number of comets. Statistical tests performed: **(C, D, I)** Two-way ANOVA with Sidak's multiple comparison test (n = 28-129 comets per genotype), **(J)** Mann-Whitney U test (n = 8-12 per genotype). ***=p≤0.001. For detailed genotypes see Supplementary Table 1 and for detailed statistics see Supplementary Table 2.

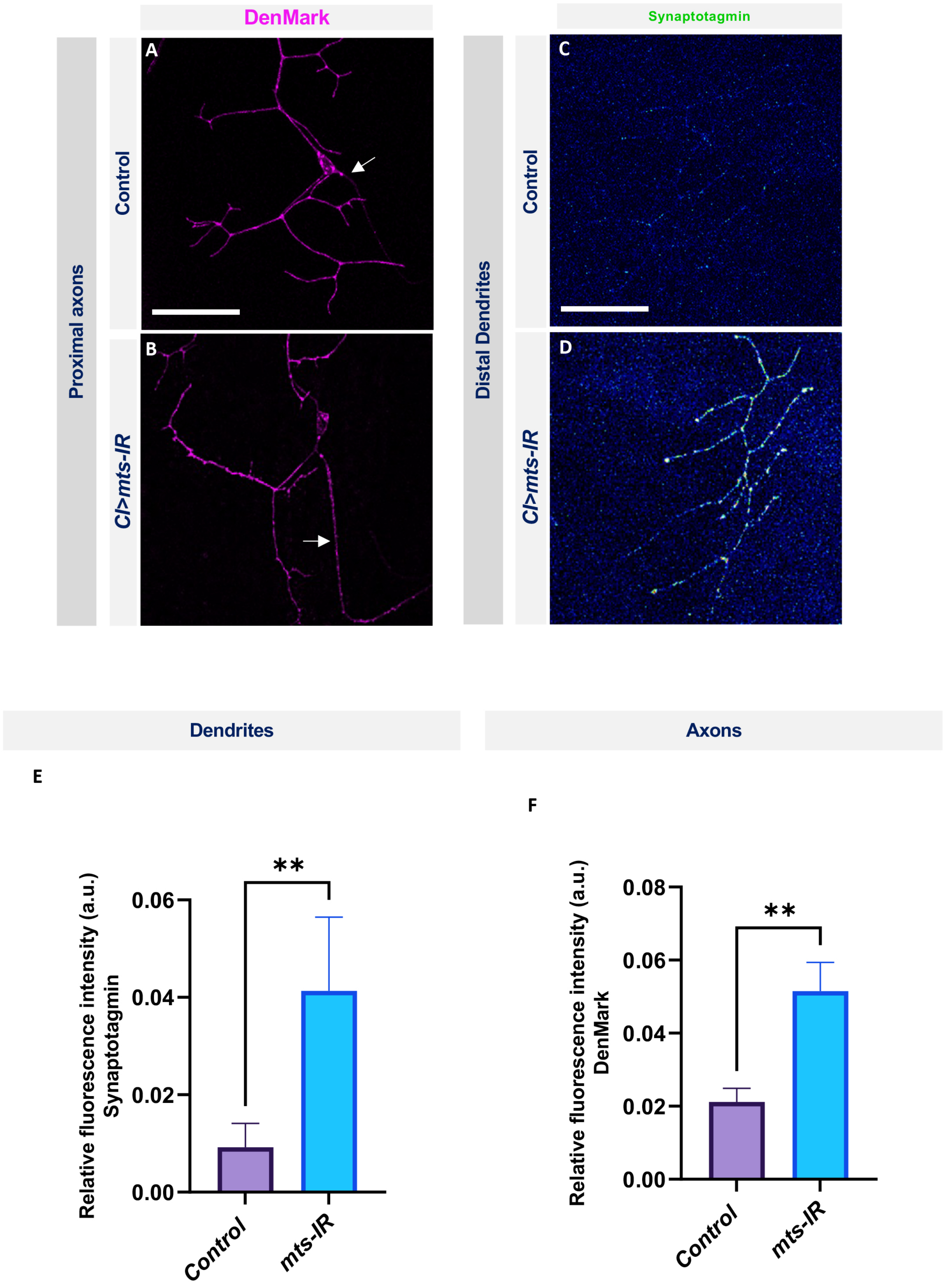
DenMark
Synaptotagmin
Proximal axons
Control
Cl>mts-IR
A
B
Distal Dendrites
Control
Cl>mts-IR
C
D
Dendrites
Axons
E
F
**
Relative fluorescence intensity (a.u.)
Synaptotagmin
0.06
0.04
0.02
0.00
Control
mts-IR
**
Relative fluorescence intensity (a.u.)
DenMark
0.08
0.06
0.04
0.02
0.00
Control
mts-IR

Supplementary Figure 7: ***mts knockdown disrupts neural compartment specification:*** **(A-D)** Representative images showing the distribution of DenMark and Synaptotagmin signal intensities in proximal axons and distal dendrites in CI neurons in **(A, C)** control and **(B, D)** *mts-IR*. **(E)** Quantitative analysis of the mean fluorescence intensities of Synaptotagmin in dendrites **(F)** Quantitative analysis of the mean fluorescence intensities of DenMark in axons. Statistical tests performed: Mann-Whitney U test **=$p \leq 0.01$ ($n = 12$-15 per genotype) Scale bar = 50μm. For detailed genotypes see Supplementary Table 1 and for detailed statistics see Supplementary Table 2.

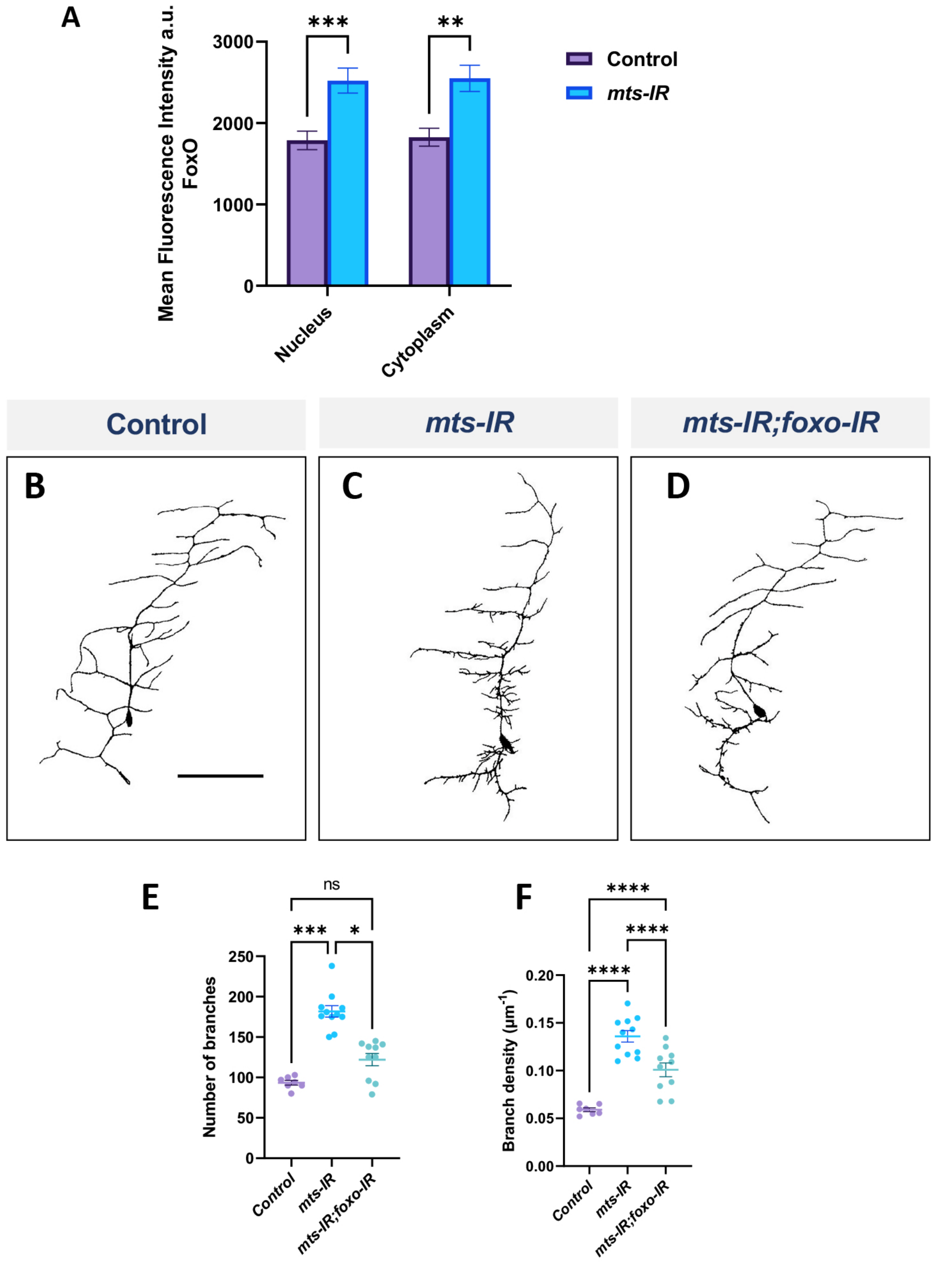

Supplementary Figure 8*: FoxO is a putative target for PP2A in CI md neurons:* **(A)** Quantitative IHC analysis showing FoxO expression in nucleus and cytoplasm in control and *mts-IR*. Representative images of **(B)** control, **(C)**, *mts-IR* **(D)** *mts-IR;foxo-IR*. **(E, F)** Quantitative morphometric analysis. Statistical tests performed: **(E)** Kruskal-Wallis with Dunn's multiple comparisons (n=7-11 per genotype). **(F)** One-way ANOVA with Sidak's multiple comparisons (n=7-11 per genotype) ***=p≤0.001, **=p≤0.01, *=p≤0.05. Scale bar = 100μm **(B)**. For detailed genotypes see Supplementary Table 1 and for detailed statistics see Supplementary Table 2.

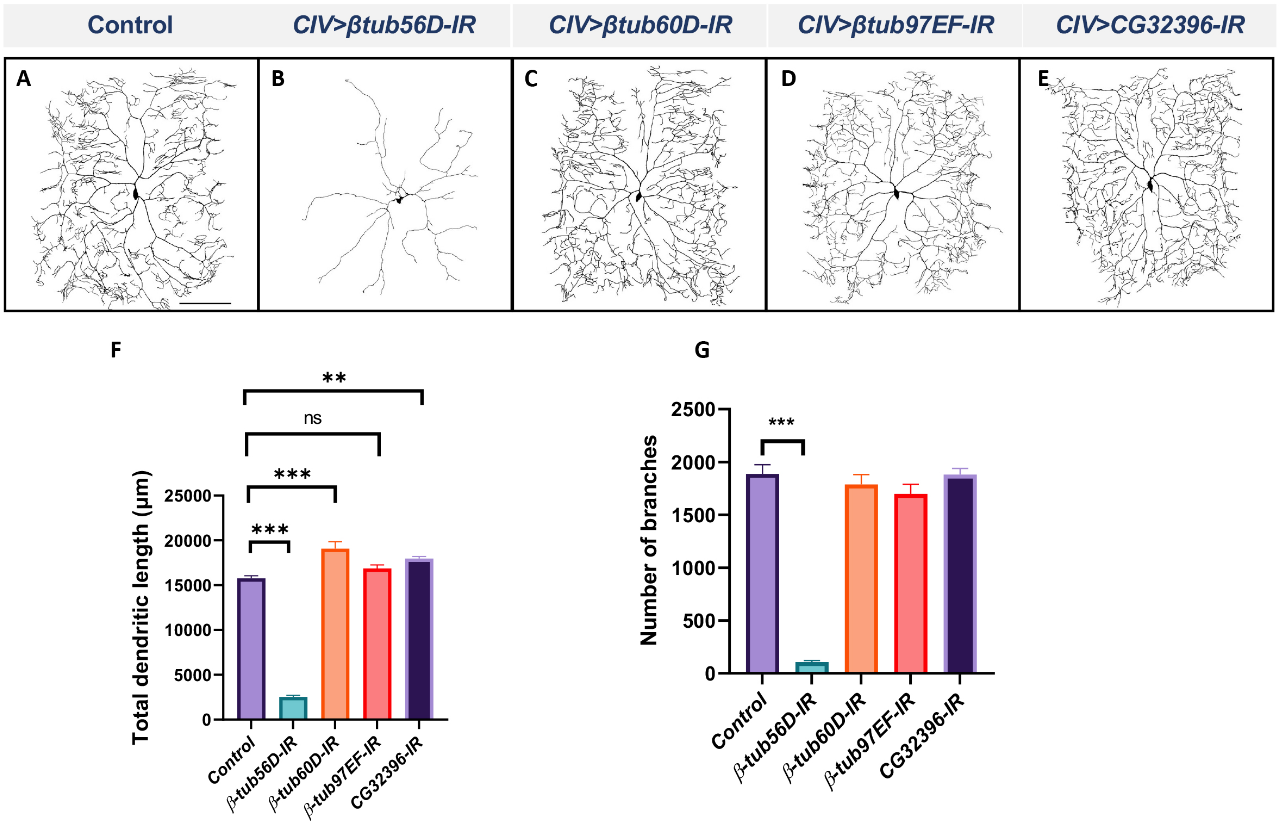

Supplementary Figure 9: ***Phenotypic analysis of β-tubulins:*** Representative images of CIV md neurons in **(A)** control, **(B)** *β-tubulin56D-IR*, **(C)** *β-tubulin60D-IR*, **(D)** *β-tubulin97EF-IR*, **(E)** *CG32396-IR*. **(F, G)** Quantitative morphometric analyses. Statistical tests performed: One-way ANOVA with Dunnet's multiple comparison test (n = 11-16 per genotype). ***=p≤0.001, **=p≤0.01. For detailed genotypes see Supplementary Table 1 and for detailed statistics see Supplementary Table 2. Scale bar = 200 μm.

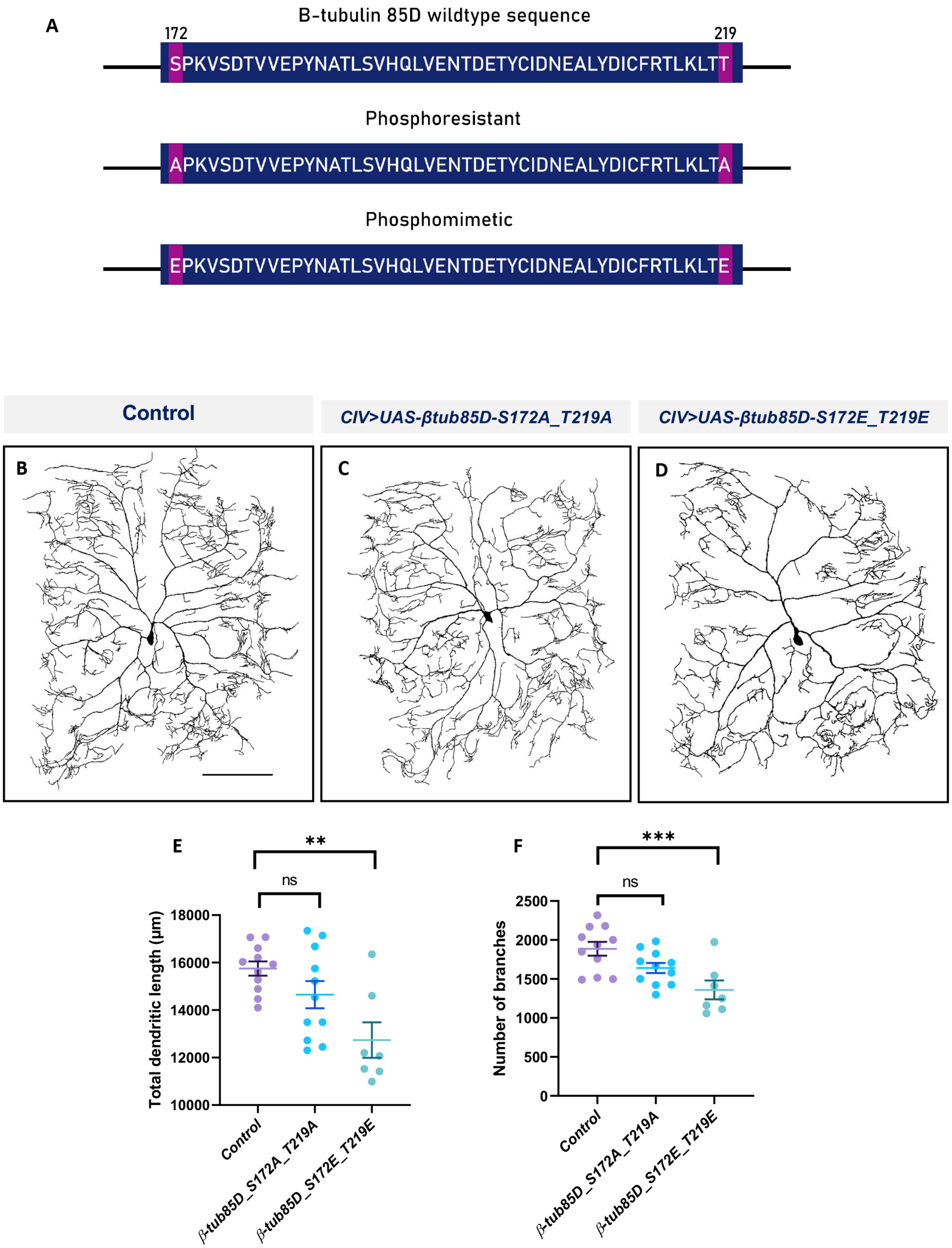
A
B-tubulin 85D wildtype sequence
172
219
SPKVSDTVVEPYNATLSVHQLVENTDETYCIDNEALYDICFRTLKLTT
Phosphoresistant
APKVSDTVVEPYNATLSVHQLVENTDETYCIDNEALYDICFRTLKLTA
Phosphomimetic
EPKVSDTVVEPYNATLSVHQLVENTDETYCIDNEALYDICFRTLKLTE
Control
CIV>UAS-βtub85D-S172A_T219A
CIV>UAS-βtub85D-S172E_T219E
B
C
D
E
**
ns
Total dendritic length (μm)
18000
16000
14000
12000
10000
Control
β-tub85D_S172A_T219A
β-tub85D_S172E_T219E
F
***
ns
Number of branches
2500
2000
1500
1000
500
0
Control
β-tub85D_S172A_T219A
β-tub85D_S172E_T219E

Supplementary Figure 10: ***Expression of phosphomimetic form of β-tubulin85D disrupts dendritic morphology:*** **(A)** Schematic representation showing the amino acids mutated in *β*-tubulin85D to generate phospho-resistant and phosphomimetic forms of the protein. Representative images of CIV neurons in **(B)** control, **(C)** *β-tubulin85D-S172A_T219A*, and **(D)** *β-tubulin85D-S172E_T219E*. **(E, F)** Quantitative morphometric analyses. Statistical tests performed: **(E, F)** One-way ANOVA with Dunnett's multiple comparison test (n = 7-11 per genotype). ***=p≤0.001, **=p≤0.01. For detailed genotypes see Supplementary Table 1 and for detailed statistics see Supplementary Table 2. Scale bar = 200 μm.

Neuron Type	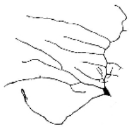 CI neurons	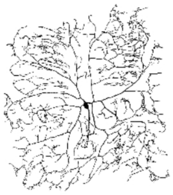 CIV neurons
Dendritic Morphology	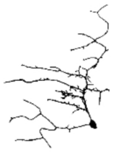 Mts restricts dendritic complexity in CI	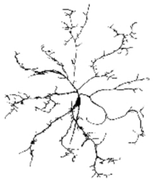 Mts promotes dendritic complexity in CIV
Effects on MT stability	Mts is required to maintain MT stability	Mts is required to maintain MT stability
Effects on MT polarity	Mts is required to maintain MT polarity	Mts is required to maintain MT polarity
Effects on F-actin	Mts is required to maintain F-actin organization	Mts is required to maintain F-actin organization and F-actin levels
Putative Mts Targets	Foxo and β-tubulin85D are putative targets of Mts in CI neurons	β-tubulin85D is a putative target of Mts in CIV neurons.

Supplementary Figure 11: ***Regulatory roles of Mts in promoting cell-type specific dendritogenesis and cytoskeletal regulation:*** Table showing the differential effects of Mts in CI and CIV md neurons.

Supplementary Table 1. Fly genotypes in each figure.

FIGURE	GENOTYPE
**1A**	*$GAL4^{477}$,UAS-mCD8::GFP/+;$GAL4^{ppk1.9}$,UAS-mCD8::GFP/+*
**1B**	*$GAL4^{477}$,UAS-mCD8::GFP/UAS-mts-IR;$GAL4^{ppk1.9}$,UAS-mCD8::GFP/+*
**1C**	*$GAL4^{5\text{-}40}$,UAS-Venus:pmSOP-$FLP^{\#42}$$FRT^{40A}$$mts^{k12502}$*
**1D**	*$GAL4^{477}$,UAS-mCD8::GFP/UAS-PP2A-29B-IR;$GAL4^{ppk1.9}$,UAS-mCD8::GFP/+*
**1N**	*$GAL4^{221}$,UAS-mCD8::GFP/+*
**1O**	*UAS-mts-IR/+;$GAL4^{221}$,UAS-mCD8::GFP/+*
**1P**	*$GAL4^{5\text{-}40}$,UAS-Venus:pmSOP-$FLP^{\#42}$$FRT^{40A}$$mts^{k12502}$*
**1Q**	*UAS-PP2A-29B-IR/+;$GAL4^{221}$,UAS-mCD8::GFP/+*
**1R**	*$GAL4^{221}$,UAS-mCD8::GFP/UAS-wd-IR*
**1S**	*$GAL4^{109(2)80}$,UAS-mCD8::GFP SOP-$FLP^{\#73}$;$FRT^{82B}$$wdb^{14}$*
**2A**	*$GAL4^{477}$,UAS-mCD8::GFP/+;$GAL4^{ppk1.9}$,UAS-mCD8::GFP/+*
**2B**	*$GAL4^{477}$,UAS-mCD8::GFP/UAS-mts;$GAL4^{ppk1.9}$,UAS-mCD8::GFP/+*

**2C**	*GAL4^{477},UAS-mCD8::GFP/UAS-PP2A-29B;GAL4$^{ppk1.9}$,UAS-mCD8::GFP/+*
**2D**	*GAL4^{477},UAS-mCD8::GFP/UAS-wdb;GAL4$^{ppk1.9}$,UAS-mCD8::GFP/+*
**2H**	*GAL4^{477},UAS-mCD8::GFP/+;GAL4$^{ppk1.9}$,UAS-mCD8::GFP/+*
**2I**	*GAL4^{477},UAS-mCD8::GFP/UAS-cka-IR;GAL4$^{ppk1.9}$,UAS-mCD8::GFP/+*
**2J**	*GAL4^{477},UAS-mCD8::GFP/+;GAL4$^{ppk1.9}$,UAS-mCD8::GFP/UAS-cka-EGFP*
**2K**	*GAL4^{477},UAS-mCD8::GFP/UAS-BFP-cka$^{\Delta PP2A}$;GAL4$^{ppk1.9}$,UAS-mCD8::GFP/+*
**3A-A'''**	*nos-GAL4/+;ppk-hCD4-tdTOMATO/+;ppk-GAL4/+*
**3 B-B'''**	*nos-GAL4/+;UAS-mts-IR/ppk-hCD4-tdTOMATO;ppl-GAL4/+*
**4A**	*GAL4^{477},UAS-mCD8::GFP/+;UAS-CD4-tdTOMATO/+*
**4B**	*GAL4^{477},UAS-mCD8::GFP/+;UAS-ct-IR/UAS-CD4-tdTOMATO*
**4C**	*GAL4^{477},UAS-mCD8::GFP/UAS-mts;UAS-ct-IR/+*
**5A-A"**	*UAS-GMA/+;GAL4^{477},UAS-mCherry::JUPITER/+*
**5B-B"**	*UAS-GMA/+;GAL4^{477},UAS-mCherry::JUPITER/UAS-mts-IR*
**5L-L"**	*UAS-GMA/+; GAL4^{221},UAS-mCherry::JUPITER/+*

**5M-M”**	*UAS-GMA/+; UAS-mts-IR/+;GAL4^{221},UAS-mCherry::JUPITER/+*
**6A-A’’’**	*GAL4^{477}/+;UAS-alphaTUB84BtdEOS/+*
**6B-B’’’**	*GAL4^{477}/UAS-mts-IR;UAS-alphaTUB84BtdEOS/+*
**6D-D’’’**	*+/+;GAL4$^{ppk1.9}$/LifeAct::tdEOS*
**6E-E’’’**	*UAS-mts-IR/+;GAL4$^{ppk1.9}$/ LifeAct::tdEOS*
**7A**	*GAL4^{ppk}/+;ppk-EB1::GFP/+*
**7B**	*GAL4^{ppk}/UAS-mts-IR;ppk-EB1::GFP/+*
**8A-A”**	*UAS-γ-tubulin23C-GFP/+;GAL4$^{ppk1.9}$,UAS-mCD8::GFP/+*
**8B-B”**	*UAS-γ-tubulin23C-GFP/UAS-mts-IR;GAL4$^{ppk1.9}$,UAS-mCD8::GFP/+*
**8D**	*UAS-Patronin-GFP/+; GAL4$^{ppk1.9}$,UAS-mCD8::RFP/+*
**8E**	*UAS-Patronin-GFP/UAS-mts-IR; GAL4$^{ppk1.9}$,UAS-mCD8::RFP/+*
**8G, G’**	*GAL4^{477},UAS-mitoGFP.AP/+;ppk-hCD4-tdTOMATO/+*
**8H, H’**	*GAL4^{477},UAS-mitoGFP.AP/UAS-mts-IR;ppk-hCD4-tdTOMATO/+*
**8I, I’**	*UAS-mitoGFP.AP/+;GAL4^{221},UAS-mCD8::RFP/+*

**8J, J’**	*UAS-mitoGFP.AP/UAS-mts-IR;GAL4^{221},UAS-mCD8::RFP/+*
**9A, A’**	*GAL4^{477},UAS-MANII-eGFP/+;ppk-hCD4-tdTOMATO/+*
**9B, B’**	*GAL4^{477},UAS-MANII-eGFP/UAS-mts-IR;ppk-hCD4-tdTOMATO/+*
**9C, C’**	*UAS-MANII-eGFP/+;GAL4^{221},UAS-mCD8::RFP/+*
**9D, D’**	*UAS-MANII-eGFP/UAS-mts-IR;GAL4^{221},UAS-mCD8::RFP/+*
**9I**	*GAL4^{477}/+;ppk-hCD4-tdTOMATO/+*
**9J**	*GAL4^{477}/UAS-mts-IR;ppk-hCD4-tdTOMATO/+*
**9K**	*GAL4^{477},UAS-mCD8::GFP/+;GAL4$^{ppk1.9}$,UAS-mCD8::GFP/+*
**9L**	*GAL4^{477},UAS-mCD8::GFP/UAS-mts-IR;GAL4$^{ppk1.9}$,UAS-mCD8::GFP/+*
**9N, N’**	*GAL4^{477},UAS-MANII-eGFP/+;ppk-hCD4-tdTOMATO/+*
**9O, O’**	*GAL4^{477},UAS-MANII-eGFP/UAS-mts-IR;ppk-hCD4-tdTOMATO/+*
**9Q, Q’**	*UAS-MANII-eGFP/+;GAL4^{221},UAS-mCD8::RFP/+*
**9R, R’**	*UAS-MANII-eGFP/UAS-mts-IR;GAL4^{221},UAS-mCD8::RFP/+*
**10A**	*GAL4^{221},UAS-mCD8::GFP/+*

**10B**	*UAS-foxo.P/+;GAL4^{221},UAS-mCD8::GFP/+*
**10C, C'**	*GAL4^{221},UAS-mCD8::GFP/+*
**10D, D'**	*UAS-mts-IR/+;GAL4^{221},UAS-mCD8::GFP/+*
**10I**	*GAL4^{221},UAS-mCD8::GFP/+*
**10J**	*UAS-mts-IR/+;GAL4^{221},UAS-mCD8::GFP/+*
**10K**	*UAS-mts/UAS-foxo.P; GAL4^{221},UAS-mCD8::GFP/+*
**11A**	*GAL4^{477},UAS-mCD8::GFP/+;GAL4$^{ppk1.9}$,UAS-mCD8::GFP/+*
**11B**	*GAL4^{477},UAS-mCD8::GFP/UAS-β-tubulin85D-IR;GAL4$^{ppk1.9}$,UAS-mCD8::GFP/+*
**11C**	*GAL4^{477},UAS-mCD8::GFP/+;GAL4$^{ppk1.9}$,UAS-mCD8::GFP/UAS-β-tubulin85D*
**11F**	*GAL4$^{ppk1.9}$,UAS-mCD::GFP/UAS-hCD4-tdTOMATO*
**11G**	*UAS-mts-IR/+;GAL4$^{ppk1.9}$,UAS-mCD::GFP/UAS-hCD4-tdTOMATO*
**11H**	*UAS-mts-IR/+;GAL4$^{ppk1.9}$,UAS-mCD::GFP/ UAS-β-tubulin85D*
**11P,P'**	*GAL4$^{ppk1.9}$,UAS-mCD::GFP/+*

**11Q,Q'**	*UAS-mts-IR/+;GAL4$^{ppk1.9}$,UAS-mCD::GFP/+*
**12A**	*GAL4^{221},UAS-mCD8::GFP/+*
**12B**	*GAL4^{221},UAS-mCD8::GFP/ UAS-β-tubulin85D-IR*
**12G**	*GAL4^{221},UAS-mCD8::GFP/+*
**12H**	*UAS-mts-IR/+;GAL4^{221},UAS-mCD8::GFP/+*
**12I**	*UAS-mts-IR/+;GAL4^{221},UAS-mCD8::GFP/ UAS-β-tubulin85D*
**S1A, A'**	*GAL4$^{ppk1.9}$,UAS-mCD8::GFP/+*
**S1B, B'**	*GAL4$^{ppk1.9}$,UAS-mCD8::GFP/+*
**S1B, B'**	*GAL4$^{ppk1.9}$,UAS-mCD8::GFP/mts-IR*
**S2C**	*GAL4^{477},UAS-mCD8::GFP/+;GAL4$^{ppk1.9}$,UAS-mCD8::GFP/+*
**S2D**	*GAL4^{477},UAS-mCD8::GFP/+;GAL4$^{ppk1.9}$,UAS-mCD8::GFP/UAS-wdb-IR*
**S2E**	*GAL4^{477},UAS-mCD8::GFP/UAS-wrd-IR;GAL4$^{ppk1.9}$,UAS-mCD8::GFP/+*
**S2F**	*GAL4^{477},UAS-mCD8::GFP/ UAS-tws-IR;GAL4$^{ppk1.9}$,UAS-mCD8::GFP/+*
**S2G**	*GAL4^{477},UAS-mCD8::GFP/UAS-CG4733-IR;GAL4$^{ppk1.9}$,UAS-mCD8::GFP/+*

**S2J**	*GAL4^{221},UAS-mCD8::GFP/+*
**S2K**	*UAS-wrd-IR/+;GAL4^{221},UAS-mCD8::GFP/+*
**S2L**	*UAS-tws-IR /+;GAL4^{221},UAS-mCD8::GFP/+*
**S2M**	*UAS-CG4733-IR/+;GAL4^{221},UAS-mCD8::GFP/+*
**S3A**	*GAL4$^{ppk1.9}$,UAS-mCD::GFP/UAS-hCD4-tdTOMATO*
**S3B**	*mts-IR/+;GAL4$^{ppk1.9}$,UAS-mCD::GFP/UAS-hCD4-tdTOMATO*
**S3C**	*mts-IR/cka-IR;GAL4$^{ppk1.9}$,UAS-mCD::GFP/+*
**S3E**	*GAL4^{477},UAS-mCD8::GFP/+;GAL4$^{ppk1.9}$,UAS-mCD8::GFP/+*
**S3F**	*GAL4^{477},UAS-mCD8::GFP/strip-IR;GAL4$^{ppk1.9}$,UAS-mCD8::GFP/+*
**S4A,A'**	*GAL4^{477},UAS-mCD8::GFP/+*
**S4B,B'**	*GAL4^{477},UAS-mCD8::GFP/UAS-mts;UAS-ct-IR/+*
**S5A**	*GAL4^{217},UAS-mCD8::GFP/+*
**S5A'**	*GAL4^{217},UAS-mCD8::GFP/UAS-mts-IR*
**S5C, C'**	*GAL4$^{ppk1.9}$,UAS-mCD8::RFP/+*

**S5D, D'**	*UAS-mts-IR/+;GAL4$^{ppk1.9}$,UAS-mCD8::RFP/+*
**S6A, G**	*GAL4^{221},UAS-EB1::GFP/+*
**S6B, H**	*UAS-mts-IR/+;GAL4^{221},UAS-EB1::GFP/+*
**S6E**	*GAL4^{ppk}/+;ppk-EB1::GFP/+*
**S6F**	*GAL4^{ppk}/UAS-mts-IR;ppk-EB1::GFP/+*
**S7A, C**	*UAS-DenMark,UAS-syn.eGFP/+;GAL4^{221}/+*
**S7B, D**	*UAS-DenMark,UAS-syn.eGFP/UAS-mts-IR;GAL4^{221}/+*
**S8 B**	*GAL4^{221},UAS-mCD8::GFP/+*
**S8 C**	*UAS-mts-IR/+;GAL4^{221},UAS-mCD8::GFP/+*
**S8 D**	*UAS-mts-IR/+;GAL4^{221},UAS-mCD8::GFP/UAS-foxo-IR*
**S9A**	*GAL4^{477},UAS-mCD8::GFP/+;GAL4$^{ppk1.9}$,UAS-mCD8::GFP/UAS-Luc-IR*
**S9B**	*GAL4^{477},UAS-mCD8::GFP/UAS-β-tubulin56D-IR;GAL4$^{ppk1.9}$,UAS-mCD8::GFP/+*
**S9C**	*GAL4^{477},UAS-mCD8::GFP/UAS-β-tubulin60D-IR;GAL4$^{ppk1.9}$,UAS-mCD8::GFP/+*

**S9D**	*GAL4*^{477}*,UAS-mCD8::GFP/UAS-β-tubulin97EF-IR;GAL4*$^{ppk1.9}$*,UAS-mCD8::GFP/+*
**S9E**	*GAL4*^{477}*,UAS-mCD8::GFP/UAS-CG32396-IR;GAL4*$^{ppk1.9}$*,UAS-mCD8::GFP/+*
**S10B**	*GAL4*^{477}*,UAS-mCD8::GFP/+;GAL4*$^{ppk1.9}$*,UAS-mCD8::GFP/ UAS-Luc-IR*
**S10C**	*GAL4*^{477}*,UAS-mCD8::GFP/+;GAL4*$^{ppk1.9}$*,UAS-mCD8::GFP/UAS-β-tubulin85D-S172A_T219A*
**S10D**	*GAL4*^{477}*,UAS-mCD8::GFP/+;GAL4*$^{ppk1.9}$*,UAS-mCD8::GFP/UAS-β-tubulin85D-S172E_T219E*

Supplementary Table 2. Detailed statistical analysis performed

Figure/Label	Passed Shapiro-Wilk Normality Test	Statistical test used	p-value	Number of neurons (N)
**Fig 1E**		**One-way ANOVA with Sidak's multiple comparison test**		
Control	Yes			10
mts-IR	Yes		<0.0001	12
PP2A-29B-IR	Yes		<0.0001	12
Control-MARCM	Yes			15
mts^{k12502}	Yes		<0.0001	12
**Fig 1F**		**One-way ANOVA with Sidak's multiple comparison test**		
Control	Yes			10
mts-IR	Yes		<0.0001	12
PP2A-29B-IR	Yes		<0.0001	12
Control-MARCM	Yes			15
mts^{k12502}	Yes		<0.0001	12
**Fig 1G**		**One-way ANOVA with Sidak's multiple comparisons**		
Control	Yes			10

mts-IR	Yes		<0.0001	12
PP2A-29B-IR	Yes		<0.0001	12
Control-MARCM	Yes		<0.0001	15
mts^{k12502}	Yes		<0.0001	12
**Fig 1H**		**Kruskal-Wallis with Dunn's multiple comparison test**		
Control	No			10
mts-IR	No		<0.0001	12
PP2A-29B-IR	No		<0.0001	12
**Fig 1I**		**Kruskal-Wallis with Dunn's multiple comparison test**		
Control-MARCM	No			15
mts^{k12502}	No		<0.0001	12
**Fig 1J** **1st order**		**Two-way ANOVA with Dunnett's test for multiple comparison**		
Control				10
mts-IR			<0.0001	12
PP2A-29B-IR			<0.0001	12
**Fig 1J** **2ndorder**		**Two-way ANOVA with Dunnett's test for multiple comparison**		

Control				10
mts-IR			<0.0001	12
PP2A-29B-IR			<0.0001	12
**Fig 1J**<br>**3rd order**		**Two-way ANOVA with Dunnett's test for multiple comparison**		
Control				10
mts-IR			<0.0001	12
PP2A-29B-IR			<0.0001	12
**Fig 1J**<br>**4th order**		**Two-way ANOVA with Dunnett's test for multiple comparison**		
Control				10
mts-IR			0.0008	12
PP2A-29B-IR			0.0522	12
**Fig 1K**<br>**5th order**		**Two-way ANOVA with Dunnett's test for multiple comparison**		
Control				10
mts-IR			<0.0001	12
PP2A-29B-IR			<0.0001	12
**Fig 1K**<br>**6th order**		**Two-way ANOVA with Dunnett's test for multiple comparison**		
Control				10

mts-IR			0.7324	12
PP2A-29B-IR			0.7531	12
**Fig 1K**<br>**7th order**		**Two-way ANOVA with Dunnett's test for multiple comparison**		
Control				10
mts-IR			0.9976	12
PP2A-29B-IR			0.9976	12
**Fig 1L**<br>**1st order**		**Two-way ANOVA with Dunnett's test for multiple comparison**		
Control-MARCM				15
mts^{k12502}			<0.0001	12
**Fig 1L**<br>**2ndorder**		**Two-way ANOVA with Dunnett's test for multiple comparison**		
Control-MARCM				15
mts^{k12502}			<0.0001	12
**Fig 1L**<br>**3rd order**		**Two-way ANOVA with Dunnett's test for multiple comparison**		
Control-MARCM				15
mts^{k12502}			<0.0001	12
**Fig 1L**<br>**4th order**		**Two-way ANOVA with Dunnett's test for multiple comparison**		

Control-MARCM				15
mts^{k12502}			0.0054	12
**Fig 1M**<br>**5th order**		**Two-way ANOVA with Dunnett's test for multiple comparison**		
Control-MARCM				15
mts^{k12502}			0.0019	12
**Fig 1M**<br>**6th order**		**Two-way ANOVA with Dunnett's test for multiple comparison**		
Control-MARCM				15
mts^{k12502}			0.7686	12
**Fig 1M**<br>**7th order**		**Two-way ANOVA with Dunnett's test for multiple comparison**		
Control-MARCM				15
mts^{k12502}			0.9942	12
**Fig 1T**		**Kruskal-Wallis with Dunn's multiple comparison test**		
Control	No			20
mts-IR	Yes		0.0007	21
PP2A-29B-IR	Yes		>0.9999	18
wdb-IR	Yes		>0.9999	18
Control-MARCM	No			34

mts^{k12502}	No		<0.0001	12
wdb^{14}	No		0.0143	12
**Fig 1U**		**Kruskal-Wallis with Dunn's multiple comparison test**		
Control	Yes			20
mts-IR	Yes		<0.0001	21
PP2A-29B-IR	Yes		<0.0001	18
wdb-IR	No		0.0176	18
Control-MARCM	No			34
mts^{k12502}	Yes		0.18390	12
wdb^{14}	Yes		>0.9999	12
**Fig 1V**		**Kruskal-Wallis with Dunn's multiple comparison test**		
Control	No			20
mts-IR	Yes		<0.0001	21
PP2A-29B-IR	Yes		<0.0001	18
wdb-IR	Yes		0.1337	18
Control-MARCM	No			34
mts^{k12502}	Yes		0.0011	12
wdb^{14}	Yes		>0.9999	12

**Fig 1W**<br>**1st order**		**Two-way ANOVA with Dunnett's test for multiple comparison**		
Control				21
mts-IR			<0.0001	20
PP2A-29B-IR			<0.0001	19
wdb-IR			0.0567	21
**Fig 1W**<br>**2nd order**		**Two-way ANOVA with Dunnett's test for multiple comparison**		
Control				21
mts-IR			0.0032	20
PP2A-29B-IR			<0.0001	19
wdb-IR			0.3571	21
**Fig 1W**<br>**3rd order**		**Two-way ANOVA with Dunnett's test for multiple comparison**		
Control				21
mts-IR			0.4371	20
PP2A-29B-IR			0.0036	19
wdb-IR			0.9226	21
**Fig 1W**<br>**4th order**		**Two-way ANOVA with Dunnett's test for multiple comparison**		
Control				21

mts-IR			0.9817	20
PP2A-29B-IR			0.8562	19
wdb-IR			0.9841	21
**Fig 1X**<br>**1st order**		**Two-way ANOVA with Dunnett's test for multiple comparison**		
Control-MARCM				33
mts^{k12502}			<0.0001	12
wdb^{14}			0.1298	12
**Fig 1X**<br>**2nd order**		**Two-way ANOVA with Dunnett's test for multiple comparison**		
Control-MARCM				33
mts^{k12502}			0.0038	12
wdb^{14}			0.7993	12
**Fig 1X**<br>**3rd order**		**Two-way ANOVA with Dunnett's test for multiple comparison**		
Control-MARCM				33
mts^{k12502}			0.0721	12
wdb^{14}			0.6939	12
**Fig 1X**<br>**4th order**		**Two-way ANOVA with Dunnett's test for multiple comparison**		

Control-MARCM				33
mts^{k12502}			0.8137	12
wdb^{14}			0.7288	12
**Fig 2E**		**One-way ANOVA with Dunnett's multiple comparison test**		
Control	Yes			9
mts-OE	Yes		<0.0001	10
PP2A-29B-OE	Yes		0.0212	14
wdb-OE	Yes		<0.0001	11
**Fig 2F**		**One-way ANOVA with Dunnett's multiple comparison test**		
Control	Yes			9
mts-OE	Yes		<0.0001	10
PP2A-29B-OE	Yes		0.0048	14
wdb-OE	Yes		0.3377	11
**Fig 2G**		**One-way ANOVA with Dunnett's multiple comparison test**		
Control	Yes			9
mts-OE	Yes		<0.0001	10
PP2A-29B-OE	Yes		0.6013	14
wdb-OE	Yes		0.0242	11

**Fig 2L**		**Kruskal-Wallis with Dunn's multiple comparison test**		
Control	Yes			10
cka-IR	Yes		<0.0001	11
cka-OE	Yes		0.0551	13
BFP-cka$^{\Delta PP2A}$	No		>0.9999	13
**Fig 2M**		**Kruskal-Wallis with Dunn's multiple comparison test**		
Control	Yes			10
cka-IR	No		0.0006	11
cka-OE	Yes		>0.9999	13
BFP-cka$^{\Delta PP2A}$	No		0.9246	13
**Fig 2N**<br>**1st order**		**Two-way ANOVA with Dunnett's test for multiple comparison**		
Control				10
cka-IR			<0.0001	11
cka-OE			0.4681	13
BFP-cka$^{\Delta PP2A}$			0.0069	13
**Fig 2N**<br>**2nd order**		**Two-way ANOVA with Dunnett's test for multiple comparison**		

Control				10
cka-IR			0.0001	11
cka-OE			0.6904	13
BFP-cka$^{\Delta PP2A}$			0.4179	13
**Fig 2N**<br>**3rd order**		**Two-way ANOVA with Dunnett's test for multiple comparison**		
Control				10
cka-IR			0.0808	11
cka-OE			0.9979	13
BFP-cka$^{\Delta PP2A}$			>0.9999	13
**Fig 2N**<br>**4th -7th order**		**Two-way ANOVA with Dunnett's test for multiple comparison**		
Control				10
cka-IR			0.4080	11
cka-OE			0.9780	13
BFP-cka$^{\Delta PP2A}$			0.3034	13
**Fig 3C**		**Two-way ANOVA with Sidak's test for multiple comparison**		
Control-24h AEL				17
mts-IR-24h AEL			>0.9999	11
Control-48h AEL				20

mts-*IR*-48h AEL			0.9998	10
Control-72h AEL				11
mts-*IR*-72h AEL			<0.0001	10
Control-96h AEL				10
mts-*IR*-96h AEL			<0.0001	9
**Fig 3D**		**Two-way ANOVA with Sidak's test for multiple comparison**		
Control-24h AEL				17
mts-*IR*-24h AEL			0.9773	11
Control-48h AEL				20
mts-*IR*-48h AEL			0.2244	10
Control-72h AEL				11
mts-*IR*-72h AEL			<0.0001	10
Control-96h AEL				10
mts-*IR*-96h AEL			<0.0001	9
**Fig 3E**		**Two-way ANOVA with Sidak's test for multiple comparison**		
Control-24h AEL				17
mts-*IR*-24h AEL			0.0762	11
Control-48h AEL				20

mts-IR-48h AEL			0.0004	10
Control-72h AEL				11
mts-IR-72h AEL			<0.0001	10
Control-96h AEL				10
mts-IR-96h AEL			<0.0001	9
**Fig 4D**		**Unpaired t-test**		
Control *(mts)*	Yes			4
ct-IR (mts)	Yes		<0.0001	4
**Fig 4E**		**One-way ANOVA with Sidak's multiple comparison test**		
Control	Yes			9
Control *vs ct-IR*	Yes		<0.0001	9, 8
ct-IR vs mts-OE;ct-IR	Yes		0.0167	8, 17
**Fig 4F**		**One-way ANOVA with Sidak's multiple comparison test**		
Control	Yes			9
Control *vs ct-IR*	Yes		<0.0001	9, 8
ct-IR vs mts-OE;ct-IR	Yes		0.0187	8, 17
**Fig 4G** **1st order**		**Two-way ANOVA with Tukey's multiple comparison test**		
Control				9

Control *vs ct-IR*			<0.0001	9, 8
ct-IR vs mts-OE;ct-IR			<0.0001	8, 17
**Fig 4G**<br>**2nd order**		**Two-way ANOVA with Tukey's multiple comparison test**		
Control				9
Control *vs ct-IR*			<0.0001	9, 8
ct-IR vs mts-OE;ct-IR			0.1024	8, 17
**Fig 4G**<br>**3rd order**		**Two-way ANOVA with Tukey's multiple comparison test**		
Control				9
Control *vs ct-IR*			<0.0001	9, 8
ct-IR vs mts-OE;ct-IR			0.8353	8, 17
**Fig 4G**<br>**4th order**		**Two-way ANOVA with Tukey's multiple comparison test**		
Control				9
Control *vs ct-IR*			0.1291	9, 8
ct-IR vs mts-OE;ct-IR			0.8353	8, 17
**Fig 4H**<br>**5th order**		**Two-way ANOVA with Tukey's multiple comparison test**		
Control				9

Control *vs ct-IR*			<0.0001	9, 8
ct-IR vs mts-OE;ct-IR			0.0648	8, 17
**Fig 4H**<br>**6th order**		**Two-way ANOVA with Tukey's multiple comparison test**		
Control				9
Control *vs ct-IR*			0.3278	9, 8
ct-IR vs mts-OE;ct-IR			0.9915	8, 17
**Fig 4H**<br>**7th order**		**Two-way ANOVA with Tukey's multiple comparison test**		
Control				9
Control *vs ct-IR*			0.9544	9, 8
ct-IR vs mts-OE;ct-IR			0.9421	8, 17
**Fig 5E**<br>**40µm from soma**		**Unpaired t-test with false discovery rate correction**		
Control				11
mts-IR			<0.0001	11
**Fig 5E**<br>**80µm from soma**		**Unpaired t-test with false discovery rate correction**		
Control				11
mts-IR			<0.0001	11

**Fig 5E**<br><br>**120µm from soma**		**Unpaired t-test with false discovery rate correction**		
Control				11
mts-IR			<0.0001	11
**Fig 5E**<br><br>**160µm from soma**		**Unpaired t-test with false discovery rate correction**		
Control				11
mts-IR			<0.0001	11
**Fig 5E**<br><br>**200µm from soma**		**Unpaired t-test with false discovery rate correction**		
Control				11
mts-IR			<0.0001	11
**Fig 5E**<br><br>**240µm from soma**		**Unpaired t-test with false discovery rate correction**		
Control				11
mts-IR			<0.0001	11
**Fig 5E**<br><br>**280µm from soma**		**Unpaired t-test with false discovery rate correction**		
Control				11
mts-IR			<0.0001	11

**Fig 5E**<br>**320µm from soma**		**Unpaired t-test with false discovery rate correction**		
Control				11
mts-IR			<0.0001	11
**Fig 5E**<br>**360µm from soma**		**Unpaired t-test with false discovery rate correction**		
Control				11
mts-IR			<0.0001	11
**Fig 5E**<br>**400µm from soma**		**Unpaired t-test with false discovery rate correction**		
Control				11
mts-IR			<0.0001	11
**Fig 5E**<br>**440µm from soma**		**Unpaired t-test with false discovery rate correction**		
Control				11
mts-IR			<0.0001	11
**Fig 5E**<br>**480µm from soma**		**Unpaired t-test with false discovery rate correction**		
Control				11
mts-IR			<0.0001	11

**Fig 5E**<br>**520µm from soma**		**Unpaired t-test with false discovery rate correction**		
Control				11
mts-IR			0.0106	11
**Fig 5E**<br>**560µm from soma**		**Unpaired t-test with false discovery rate correction**		
Control				11
mts-IR			0.1065	11
**Fig 5E**<br>**600µm from soma**		**Unpaired t-test with false discovery rate correction**		
Control				11
mts-IR			0.1140	11
**Fig 5E**<br>**640µm from soma**		**Unpaired t-test with false discovery rate correction**		
Control				11
mts-IR			0.1502	11
**Fig 5F**<br>**1st order**		**Unpaired t-test with false discovery rate correction**		
Control				11
mts-IR			0.0058	11

**Fig 5F**<br>**2nd order**		**Unpaired t-test with false discovery rate correction**		
Control				11
mts-IR			<0.0001	11
**Fig 5F**<br>**3rd order**		**Unpaired t-test with false discovery rate correction**		
Control				11
mts-IR			<0.0001	11
**Fig 5F**<br>**4th order**		**Unpaired t-test with false discovery rate correction**		
Control				11
mts-IR			<0.0001	11
**Fig 5F**<br>**5th order**		**Unpaired t-test with false discovery rate correction**		
Control				11
mts-IR			<0.0001	11
**Fig 5G**<br>**40µm from soma**		**Unpaired t-test with false discovery rate correction**		
Control				11
mts-IR			0.1732	11

**Fig 5G**<br><br>**80µm from soma**		**Unpaired t-test with false discovery rate correction**		
Control				11
mts-IR			0.1114	11
**Fig 5G**<br><br>**120µm from soma**		**Unpaired t-test with false discovery rate correction**		
Control				11
mts-IR			0.0582	11
**Fig 5G**<br><br>**160µm from soma**		**Unpaired t-test with false discovery rate correction**		
Control				11
mts-IR			0.0582	11
**Fig 5G**<br><br>**200µm from soma**		**Unpaired t-test with false discovery rate correction**		
Control				11
mts-IR			0.0497	11
**Fig 5G**<br><br>**240µm from soma**		**Unpaired t-test with false discovery rate correction**		
Control				11
mts-IR			0.1732	11

**Fig 5G**<br>**280µm from soma**		**Unpaired t-test with false discovery rate correction**		
Control				11
mts-IR			0.0727	11
**Fig 5G**<br>**320µm from soma**		**Unpaired t-test with false discovery rate correction**		
Control				11
mts-IR			<0.0001	11
**Fig 5G**<br>**360µm from soma**		**Unpaired t-test with false discovery rate correction**		
Control				11
mts-IR			<0.0001	11
**Fig 5G**<br>**400µm from soma**		**Unpaired t-test with false discovery rate correction**		
Control				11
mts-IR			<0.0001	11
**Fig 5G**<br>**440µm from soma**		**Unpaired t-test with false discovery rate correction**		
Control				11
mts-IR			<0.0001	11

**Fig 5G**<br>**480µm from soma**		**Unpaired t-test with false discovery rate correction**		
Control				11
mts-IR			0.0025	11
**Fig 5G**<br>**520µm from soma**		**Unpaired t-test with false discovery rate correction**		
Control				11
mts-IR			0.0510	11
**Fig 5G**<br>**560µm from so**ma		**Unpaired t-test with false discovery rate correction**		
Control				11
mts-IR			0.0582	11
**Fig 5G**<br>**600µm from soma**		**Unpaired t-test with false discovery rate correction**		
Control				11
mts-IR			0.2530	11
**Fig 5G**<br>**640µm from soma**		**Unpaired t-test with false discovery rate correction**		
Control				11
mts-IR			0.4808	11
**Fig 5H**		**Unpaired t-test**		

Control	Yes			11
mts-IR	Yes		<0.0001	11
**Fig 5I**		**Unpaired t-test**		
Control	Yes			11
mts-IR	Yes		<0.0001	11
**Fig 5J**<br>**1st order**		**Mann-Whitney test with FDR correction**		
Control				11
mts-IR			<0.0001	11
**Fig 5J**<br>**2nd order**		**Mann-Whitney test with FDR correction**		
Control				11
mts-IR			<0.0001	11
**Fig 5J**<br>**3rd order**		**Mann-Whitney test with FDR correction**		
Control				11
mts-IR			<0.0001	11
**Fig 5J**<br>**4th order**		**Mann-Whitney test with FDR correction**		
Control				11
mts-IR			<0.0001	11

**Fig 5J**<br>**5th order**		**Mann-Whitney test with FDR correction**		
Control				11
mts-IR			0.0169	11
**Fig 5J**<br>**6th order**		**Mann-Whitney test with FDR correction**		
Control				11
mts-IR			0.0004	11
**Fig 5O**		**Unpaired t-test**		
Control				8
mts-IR			<0.0001	9
**Fig 5P**<br>**1st order**		**Unpaired t-test with false discovery rate correction**		
Control				8
mts-IR			<0.0001	9
**Fig 5P**<br>**2nd order**		**Unpaired t-test with false discovery rate correction**		
Control				8
mts-IR			<0.0001	9

**Fig 5P**<br>**3rd order**		**Unpaired t-test with false discovery rate correction**		
Control				8
mts-IR			0.0001	9
**Fig 5P**<br>**4th order**		**Unpaired t-test with false discovery rate correction**		
Control				8
mts-IR			0.0384	9
**Fig 5Q**		**Unpaired t-test**		
Control				8
mts-IR			0.0002	9
**Fig 5R**<br>**1st order**		**Mann-Whitney test with FDR correction**		
Control				8
mts-IR			0.0002	9
**Fig 5R**<br>**2nd order**		**Mann-Whitney test with FDR correction**		
Control				8
mts-IR			0.0003	9
**Fig 5R**		**Mann-Whitney test with FDR correction**		

**3rd order**				
Control				8
mts-IR			0.03646	9
**Fig 5R**<br>**4th order**		**Mann-Whitney test with FDR correction**		
Control				8
mts-IR			0.3813	9
**Fig 5S**		**Mann-Whitney test**		
Control	No			8
mts-IR	No		0.0498	9
**Fig 6C**<br>**0 min**		**Two-way ANOVA with Sidak's multiple comparison test**		
Control				11
mts-IR			>0.9999	8
**Fig 6C**<br>**30 min**		**Two-way ANOVA with Sidak's multiple comparison test**		
Control				11
mts-IR			0.0001	8
**Fig 6C**<br>**60 min**		**Two-way ANOVA with Sidak's multiple comparison test**		

Control				11
mts-IR			0.0028	8
**Fig 6F**<br>**0 min**		**Two-way ANOVA with Sidak's multiple comparison test**		
Control				13
mts-IR			>0.9999	10
**Fig 6F**<br>**30 min**		**Two-way ANOVA with Sidak's multiple comparison test**		
Control				13
mts-IR			0.0006	10
**Fig 6F**<br>**60 min**		**Two-way ANOVA with Sidak's multiple comparison test**		
Control				13
mts-IR			<0.0001	10
**Fig 7C**<br>**Retrograde**		**Two-way ANOVA with Sidak 's multiple comparison test**		
Control				72<br>(comets)
mts-IR			<0.0001	70<br>(comets)

**Fig 7C**<br>**Anterograde**		**Two-way ANOVA with Sidak 's multiple comparison test**		
Control				72 (comets)
mts-IR			<0.0001	70 (comets)
**Fig 7D**		**Mann Whitney test**		
Control	No			13
mts-IR	Yes		<0.0001	9
**Fig 7E**<br>**Retrograde**		**Two-way ANOVA with Dunnett's multiple comparison test**		
Control				54 (comets)
mts-IR			0.0074	43 (comets)
**Fig 7E**<br>**Anterograde**		**Two-way ANOVA with Dunnett's multiple comparison test**		
Control				54 (comets)
mts-IR			0.0074	43 (comets)
**Fig 7F**		**Unpaired t-test**		
Control	Yes			14
mts-IR	Yes		0.9595	9
**Fig 7G**		**Mann-Whitney test**		
Control	No			60 (comets)

mts-IR	Yes		0.8499	29 (comets)
**Fig 7H**		**Mann-Whitney test**		
Control	No			67 (comets)
mts-IR	Yes		0.0001	36 (comets)
**Fig 7I**		**Mann-Whitney test**		
Control	No			27 (comets)
mts-IR	No		0.4224	19 (comets)
**Fig 7J**		**Mann-Whitney test**		
Control	No			34 (comets)
mts-IR	No		0.0312	26 (comets)
**Fig 7K**<br>**CI Anterograde**		**Two-way ANOVA with Dunnett's multiple comparison test**		
Control	Yes			42 (comets)
mts-IR	Yes		<0.0001	53 (comets)
**Fig 7K**<br>**CI Retrograde**		**Two-way ANOVA with Dunnett's multiple comparison test**		
Control	Yes			42 (comets)
mts-IR	Yes		<0.0001	53 (comets)
**Fig 7L**		**Mann-Whitney test**		
Control	No			52 (comets)
mts-IR	No		<0.0001	67(comets)

**Fig 7M**		**Mann-Whitney test**		
Control	No			15
mts-IR	Yes		<0.0001	9
**Fig 7N**		**Mann-Whitney test**		
Control	No			52 (comets)
mts-IR	No		0.1145	67(comets)
**Fig 8C**		**Unpaired t-test**		
Control	Yes			10
mts-IR	Yes		<0.0001	10
**Fig 8F**		**Mann-Whitney test**		
Control	Yes			8
mts-IR	No		0.0062	10
**Fig 8K**		**Unpaired t-test**		
Control	Yes			10
mts-IR	Yes		<0.0001	11
**Fig 8L**		**Unpaired t-test**		
Control	Yes			10
mts-IR	Yes		<0.0001	11
**Fig 8M**		**Unpaired t-test**		
Control	Yes			7

mts-IR	Yes		0.0004	14
**Fig 8N**		**Unpaired t-test**		
Control	Yes			7
mts-IR	Yes		0.0002	14
**Fig 9E**		**Unpaired t-test**		
Control	Yes			19
mts-IR	Yes		<0.0001	13
**Fig 9F**		**Unpaired t-test**		
Control	Yes			19
mts-IR	Yes		<0.0001	13
**Fig 9G**		**Unpaired t-test**		
Control	Yes			10
mts-IR	Yes		0.0357	12
**Fig 9H**		**Mann-Whitney test**		
Control	No			10
mts-IR	Yes		0.6676	11
**Fig 9P**		**Mann-Whitney test**		
Control	No			18
mts-IR	No		<0.0001	12
**Fig 9S**		**Unpaired t-test**		

Control	Yes			10
mts-IR	Yes		0.0025	12
**Fig 10E**		**Unpaired t-test**		
Control	Yes			23
mts-IR	Yes		0.0005	29
**Fig 10F**		**Unpaired t-test**		
Control	Yes			10
foxo-OE	Yes		0.0042	12
**Fig 10G**		**Unpaired t-test**		
Control	Yes			10
foxo-OE	Yes		0.0003	12
**Fig 10H**		**Unpaired t-test**		
Control	Yes			10
foxo-OE	Yes		<0.0001	12
**Fig 10M**		**One-way ANOVA with Sidak's multiple comparison test**		
Control	Yes			10
Control *vs mts-OE/foxo-OE*	Yes		0.0113	10, 14
mts-IR vs mts-OE/foxo-OE	Yes		<0.0001	12, 14

**Fig 10N**		**One-way ANOVA with Sidak's multiple comparison test**		
Control	Yes			10
Control *vs mts-OE/foxo-OE*	Yes		0.1984	10, 14
mts-IR vs mts-OE/foxo-OE	Yes		<0.0001	12, 14
**Fig 11D**		**One-way ANOVA with Dunnett's multiple comparison test**		
Control	Yes			13
β-tubulin 85D-IR	Yes		<0.0001	13
β-tubulin 85D-OE	Yes		0.9620	13
**Fig 11E**		**One-way ANOVA with Dunnett's multiple comparison test**		
Control	Yes			13
β-tubulin 85D-IR	Yes		<0.0001	13
β-tubulin 85D-OE	Yes		0.3584	13
**Fig 11I**		**Kruskal-Wallis with Dunn's multiple comparison test**		
Control	Yes			10
Control *vs mts-IR*	No		<0.0001	10, 11
mts-IR vs mts-IR; β-tubulin 85D-OE	Yes		0.0451	11, 12

**Fig 11J**		**Kruskal-Wallis with Dunn's multiple comparison test**		
Control	Yes			10
Control *vs mts-IR*	No		<0.0001	10, 11
mts-IR vs mts-IR; β-tubulin 85D-OE	Yes		0.0290	11, 12
**Fig 11K**		**One-way ANOVA with Sidak's multiple comparison test**		
Control	Yes			10
Control *vs mts-IR*	Yes		<0.0001	10, 11
mts-IR vs mts-IR; β-tubulin 85D-OE	Yes		0.0001	11, 12
**Fig 11L** **1st order**		**Two-way ANOVA with Tukey's multiple comparison test**		
Control				10
Control *vs mts-IR*			<0.0001	10, 11
mts-IR vs mts-IR; β-tubulin 85D-OE			<0.0001	11, 12
**Fig 11L** **2nd order**		**Two-way ANOVA with Tukey's multiple comparison test**		
Control				10
Control *vs mts-IR*			<0.0001	10, 11

mts-IR vs mts-IR; β-tubulin 85D-OE			<0.0001	11, 12
**Fig 11L**<br>**3rd order**		**Two-way ANOVA with Tukey's multiple comparison test**		
Control				10
Control *vs mts-IR*			<0.0001	10, 11
mts-IR vs mts-IR; β-tubulin 85D-OE			0.1186	11, 12
**Fig 11L**<br>**4th order**		**Two-way ANOVA with Tukey's multiple comparison test**		
Control				10
Control *vs mts-IR*			0.0006	10, 11
mts-IR vs mts-IR; β-tubulin 85D-OE			0.3313	11, 12
**Fig 11L**<br>**5th order**		**Two-way ANOVA with Tukey's multiple comparison test**		
Control				10
Control *vs mts-IR*			0.6880	10, 11
mts-IR vs mts-IR; β-tubulin 85D-OE			0.9638	11, 12
**Fig 11M**<br>**6th order**		**Two-way ANOVA with Tukey's multiple comparison test**		
Control				10

Control *vs mts-IR*			0.1067	10, 11
mts-IR vs mts-IR; β-tubulin 85D-OE			0.7650	11, 12
**Fig 11M**<br>**7th order**		**Two-way ANOVA with Tukey's multiple comparison test**		
Control				10
Control *vs mts-IR*			0.9247	10, 11
mts-IR vs mts-IR; β-tubulin 85D-OE			0.9961	11, 12
**Fig 11N**		**One-way ANOVA with Sidak's multiple comparison test**		
Control	Yes			10
Control *vs mts-IR*	Yes		<0.0001	10, 11
mts-IR vs mts-IR; β-tubulin 85D-OE	Yes		<0.0001	11, 12
**Fig 11R**		**Unpaired t-test**		
Control	Yes			24
mts-IR	Yes		<0.0001	32
**Fig 12C**		**Unpaired t-test**		
Control	Yes			10
β-tubulin 85D-IR	Yes		0.0522	19
**Fig 12D**		**Unpaired t-test**		

Control	Yes			10
β-tubulin 85D-IR	Yes		0.0360	19
**Fig 12E**		**Unpaired t-test**		
Control	Yes			10
β-tubulin 85D-IR	Yes		<0.0001	19
**Fig 12F**<br>**1st order**		**Unpaired t-test with FDR correction**		
Control	Yes			10
β-tubulin 85D-IR	Yes		0.0072	19
**Fig 12F**<br>**2nd order**		**Unpaired t-test with FDR correction**		
Control	Yes			10
β-tubulin 85D-IR	Yes		0.0015	19
**Fig 12F**<br>**3rd order**		**Unpaired t-test with FDR correction**		
Control	Yes			10
β-tubulin 85D-IR	Yes		0.5066	19
**Fig 12F**<br>**4th order**		**Unpaired t-test with FDR correction**		
Control	Yes			10
β-tubulin 85D-IR	Yes		0.2024	19

**Fig 12K**		**One-way ANOVA with Sidak's multiple comparison test**		
Control	Yes			10
Control *vs mts-IR;β-tubulin 85D-OE*	Yes		0.0338	10, 19
mts-IR vs mts-IR;β-tubulin 85D-OE	Yes		<0.0001	14, 19
**Fig 12L**		**One-way ANOVA with Sidak's multiple comparison test**		
Control	Yes			10
Control *vs mts-IR;β-tubulin 85D-OE*	Yes		0.0003	10, 19
mts-IR vs mts-IR;β-tubulin 85D-OE	Yes		0.0037	14, 19
**Fig 12M**<br>**1st order**		**Two-way ANOVA with Tukey's multiple comparison test**		
Control	Yes			10
Control *vs mts-IR;β-tubulin 85D-OE*	Yes		0.0060	10, 19
mts-IR vs mts-IR;β-tubulin 85D-OE	Yes		<0.0001	14, 19
**Fig 12M**<br>**2nd order**		**Two-way ANOVA with Tukey's multiple comparison test**		
Control	Yes			10

Control *vs mts-IR;β-tubulin 85D-OE*	Yes		0.0213	10, 19
mts-IR vs mts-IR;β-tubulin 85D-OE	Yes		0.0046	14, 19
**Fig 12M** **3rd order**		**Two-way ANOVA with Tukey's multiple comparison test**		
Control				10
Control *vs mts-IR;β-tubulin 85D-OE*	Yes		0.9899	10, 19
mts-IR vs mts-IR;β-tubulin 85D-OE	Yes		0.0181	14, 19
**Fig 12M** **4th order**		**Two-way ANOVA with Tukey's multiple comparison test**		
Control				10
Control *vs mts-IR;β-tubulin 85D-OE*	Yes		0.9658	10, 19
mts-IR vs mts-IR;β-tubulin 85D-OE	Yes		0.6491	14, 19

SUPPLEMENTALS

**Fig S1D**		**Unpaired t-test**		
Control				18
mts-IR	Yes		0.0434	5

**Fig S2A**		**Kruskal-Wallis with Dunn's multiple comparison test**		
Control	Yes			10
mts-IR	No		<0.0001	12
PP2A-29B-IR	Yes		0.0002	12
Control-MARCM	Yes			15
mts^{k12502}	Yes		<0.0001	12
**Fig S2B**		**Kruskal-Wallis with Dunn's multiple comparison test**		
Control	Yes			10
mts-IR	No		0.1769	12
PP2A-29B-IR	Yes		0.0018	12
Control-MARCM	Yes			15
mts^{k12502}	Yes		<0.0001	12
**Fig S2H**		**One-way ANOVA with Sidak's multiple comparison test**		
Control	Yes			10
wdb-IR	Yes		0.0082	13
wrd-IR	Yes		0.0212	12
tws-IR	Yes		0.0192	16

CG4733-IR	Yes		0.1024	12
**Fig S2I**		**Kruskal-Wallis with Dunn's multiple comparison test**		
Control	Yes			10
wdb-IR	Yes		0.0002	13
wrd-IR	No		0.4980	12
tws-IR	Yes		0.0522	16
CG4733-IR	Yes		0.6844	12
**Fig S2N**		**Kruskal-Wallis with Dunn's multiple comparison test**		
Control	Yes			10
wrd-IR	Yes		0.9407	11
tws-IR	No		>0.9999	10
CG4733-IR	No		0.0278	16
**Fig S2O**		**Kruskal-Wallis with Dunn's multiple comparison test**		
Control	Yes			10
wrd-IR	Yes		>0.9999	11
tws-IR	Yes		>0.9999	10
CG4733-IR	No		0.4583	16

**Fig S3D**		**One-way ANOVA with Sidak's multiple comparison test**		
Control	Yes			10
Control vs *mts-IR*	Yes		<0.0001	11
mts-IR vs *mts-IR;cka-IR*	Yes		>0.9999	9
Control vs *mts-IR;cka-IR*	Yes		<0.0001	9
**Fig S3G**		**Unpaired t-test**		
Control	Yes			9
strip-IR	Yes		0.0300	18
**Fig S3H**		**Unpaired t-test**		
Control	Yes			9
strip-IR	Yes		0.2104	18
**Fig S4C**		**Unpaired t-test**		
Control	Yes			13
mts-OE;ct-IR	Yes		0.1418	8
**Fig S5B**<br>**CI**		**Two-way ANOVA with Dunnett's multiple comparison test**		
Control				14
mts-IR			<0.0001	18

**Fig S5B**<br><br>**CIII**		**Two-way ANOVA with Dunnett's multiple comparison test**		
Control				14
mts-IR			<0.0001	18
**Fig S5B**<br><br>**CIV**		**Two-way ANOVA with Dunnett's multiple comparison test**		
Control				14
mts-IR			0.0007	18
**Fig S5E**		**Unpaired t-test**		
Control				11
mts-IR			0.0427	16
**Fig S6C**<br><br>**CI Anterograde**		**Two-way ANOVA with Sidak's multiple comparison test**		
Control				53 (comets)
mts-IR			0.6881	35 (comets)
**Fig S6C**<br><br>**CI Retrograde**		**Two-way ANOVA with Sidak's multiple comparison test**		
Control				53 (comets)
mts-IR			0.6881	35 (comets)
**Fig S6D**<br><br>**CIV Anterograde**		**Two-way ANOVA with Sidak's multiple comparison test**		

Control				38 (comets)
mts-IR			0.9237	28 (comets)
**Fig S6D**<br>**CIV Retrograde**		**Two-way ANOVA with Sidak's multiple comparison test**		
Control				38 (comets)
mts-IR			0.9237	28 (comets)
**Fig S6I**<br>**CI Anterograde**		**Two-way ANOVA with Sidak's multiple comparison test**		
Control				49 (comets)
mts-IR			0.9237	129 (comets)
**Fig S6I**<br>**CI Retrograde**		**Two-way ANOVA with Sidak's multiple comparison test**		
Control				49 (comets)
mts-IR			0.9237	129 (comets)
**Fig S6J**		**Mann-Whitney test**		
Control	Yes			12
mts-IR	No		<0.0001	8
**Fig S7E**		**Mann-Whitney test**		
Control	No			12
mts-IR	No		0.0070	15

**Fig S7F**		**Mann-Whitney test**		
Control	No			12
mts-IR	Yes		0.0087	15
**Fig S8A Nucleus**		**Two-way ANOVA with Sidak's multiple comparison test**		
Control	Yes			23
mts-IR	Yes		0.0010	23
**Fig S8A Cytoplasm**		**Two-way ANOVA with Sidak's multiple comparison test**		
Control	Yes			23
mts-IR	Yes		0.0012	23
**Fig S8E**		**Kruskal-Wallis with Dunn's multiple comparison test**		
Control	Yes			7
mts-IR	Yes		0.0009	11
mts-IR;foxo-IR	No		0.0270	10
**FigS8F**		**One-way ANOVA with Sidak's multiple**		
Control	Yes			7
mts-IR	Yes		<0.0001	11
mts-IR;foxo-IR	Yes		<0.0001	10

**Fig S9F**		**One-way ANOVA with Sidak's multiple comparison test**		
Control	Yes			11
β-tubulin 56D-IR	Yes		<0.0001	10
β-tubulin 60D-IR	Yes		<0.0001	10
β-tubulin 97EF-IR	Yes		0.2713	16
CG32396-IR	Yes		0.0065	9
**Fig S9G**		**One-way ANOVA with Dunnett's multiple comparison test**		
Control	Yes			
β-tubulin 56D-IR	Yes		<0.0001	10
β-tubulin 60D-IR	Yes		0.8552	10
β-tubulin 97EF-IR	Yes		0.2081	16
CG32396-IR	Yes		>0.9999	9
**Fig S10E**		**One-way ANOVA with Dunnett's multiple comparison test**		
Control	Yes			11
β-tubulin_S172A_T219A	Yes		0.2192	11
β-tubulin_S172E_T219E	Yes		0.0015	7

**Fig S10F**		**One-way ANOVA with Dunnett's multiple comparison test**		
Control	Yes			11
β-tubulin_S172A_T219A	Yes		0.0809	11
β-tubulin_S172E_T219E	Yes		0.0010	7

Supplementary Table S3

LIST OF GENETIC STRAINS USED IN THIS STUDY

NO.	Gene Symbol	Stock number/Source
**1.**	*mts-IR*	**B57034**
**2.**	*mts-IR*	B27723
**3.**	*mts-IR*	B38337
**4.**	*PP2A-29B-IR*	**B50533**
**5.**	*PP2A-29B-IR*	B29384
**6.**	*PP2A-29B-IR*	B43283
**7.**	*PP2A-29B-IR*	v49672
**8.**	*wdb-IR*	**B38950**
**9.**	*wdb-IR*	B38901
**10.**	*wdb-IR*	v27470
**11.**	*wdb-IR*	v101406
**12.**	*wrd-IR*	**B38900**

**13.**	*wrd-IR*	B30512
**14.**	*wrd-IR*	v22614
**15.**	*wrd-IR*	v22615
**16.**	*tws-IR*	**B58053**
**17.**	*tws-IR*	B28714
**18.**	*tws-IR*	B36698
**19.**	*tws-IR*	v34340
**20.**	*tws-IR*	v104167
**21.**	*CG4733-IR*	**v34894**
**22.**	*CG4733-IR*	v107621
**23.**	*cka-IR*	**v35234**
**24.**	*cka-IR*	v35232
**25.**	*β-tubulin85D-IR*	**B65163**
**25.**	*β-tubulin56D-IR*	**B65028**
**26.**	*β-tubulin60D-IR*	**B65856**
**27.**	*β-tubulin97EF-IR*	**B64858**
**28.**	*CG32396-IR*	**B68474**
**29.**	*UAS-mts*	**B53709**
**30.**	*strip-IR*	**v106184**
**31.**	*foxo-IR*	**B25997**

	Overexpression lines	
**32.**	*UAS-PP2A-29B*	**B55049**
**33.**	*UAS-PP2A-29B*	B55048
**34.**	*UAS-PP2A-29B*	B55050
**35.**	*UAS-wdb*	**B55052**
**36.**	*UAS-wdb*	B55051
**37.**	*UAS-cka-eGFP*	**B53757**
**38.**	*UAS-cka-eGFP*	B53786
**39.**	*UAS-foxo*	**B9575**
**40.**	*UAS-foxo*	**B42221**
**41.**	*UAS-β-tubulin85D*	**F001711**
	Mutant lines	
**42.**	mts^{k12502}	**111466 Kyoto Stock Center**
**43.**	wdb^{14}	**B53712**
**44.**	$UAS\text{-}BFP\text{-}cka^{\Delta PP2A}$	(Neisch *et al*, 2017)
	Additional fly lines used	
**45.**	*$GAL4^{477}$,UASmCD8::GFP/CyO,tubP-GAL80;$GAL4^{ppk.1.9}$,UAS-mCD8::GFP (CIV-GAL4)*	
**46.**	*$GAL4^{221}$, UAS-mCD8::GFP (CI-GAL4)*	
**47.**	*$GAL^{5\text{-}40}$UAS-Venus:pm SOP-FLP#42;tubP-GAL80FRT40A (2L MARCM)*	(Shimono *et al*, 2014)

**48.**	*hsFLP-UASmCD8:GFP; GAL4$^{109(2)80}$UAS-mCD8::GFP SOP-FLP$^{\#73}$/CyO; FRT82B tub-GAL80 (3R MARCM)*	(Shimono *et al*, 2014)
**49.**	*GAL4^{477};ppk-hCD4::tdTomato, ppk-CD8-eGFP*	
**50.**	*GAL4^{Nanos};+;ppk-::tdTomato*	**B7303**
**51.**	*GAL4^{477},UAS-mCD8::GFP;UAS-Ct-IR/TM3,Ser*	
**52.**	*UAS-GMA;GAL4^{477},UAS-mCherry::JUPITER*	(Das *et al*, 2017)
**53.**	*UAS-GMA;+:GAL4^{221},UAS-mCherry::JUPITER*	(Das *et al*, 2017)
**54.**	*UAS-alphaTUB84B.tdEOS*	**B51314**
**55.**	*UAS-LifeAct.tdEOS*	This study
**56.**	*GAL4^{ppk}/CyO;ppk-EB1::GFP*	**Gift from Dr. Jill Wildonger**
**57.**	*UAS-EB1::GFP*	**B35512**
**58.**	*UAS-γ-tubulin23C-GFP*	**Gift from Dr. Melissa Rolls**
**59.**	*UAS-YFP-Patronin*	(Feng *et al*, 2019)
**60.**	*UAS-mitoGFP.AP*	**B8442**
**61.**	*GAL4^{477},UAS-MANII-eGFP;ppk-hCD4-tdTOMATO*	
**62.**	*UAS-MANII-eGFP;GAL4^{221},UAS-mCD8::RFP*	
**63.**	*UAS-CD4-tdTom*	**B35837**
**64.**	*UAS-Luc-IR*	**B31603**
**65.**	*UAS-DenMark, UAS-syt.eGFP*	**B33064**

**66.**	*UAS-βtubulin85D_S172A_219A*	This study
**67.**	*UAS-βtubulin85D_S172E_219E*	This study
**68.**	*OregonR(ORR) (control strain)*	

Stock numbers refers to either Bloomington Stock Center or if it starts with 'v', it refers to Vienna Drosophila Research Centre; F refers to FlyORF. Stock numbers of the genes for which representative data are presented in this paper are highlighted in **bold.**

Das R, Bhattacharjee S, Patel AA, Harris JM, Bhattacharya S, Letcher JM, Clark SG, Nanda S, Iyer EPR, Ascoli GA, *et al* (2017) Dendritic Cytoskeletal Architecture Is Modulated by Combinatorial Transcriptional Regulation in Drosophila melanogaster. *Genetics* 207: genetics.300393.2017

Feng C, Thyagarajan P, Shorey M, Seebold DY, Weiner AT, Albertson RM, Rao KS, Sagasti A, Goetschius DJ & Rolls MM (2019) Patronin-mediated minus end growth is required for dendritic microtubule polarity. *J Cell Biol* 218: 2309–2328

Neisch AL, Neufeld TP & Hays TS (2017) A STRIPAK complex mediates axonal transport of autophagosomes and dense core vesicles through PP2A regulation. *J Cell Biol* 216: 441–461

Shimono K, Fujishima K, Nomura T, Ohashi M, Usui T, Kengaku M, Toyoda A & Uemura T (2014) An evolutionarily conserved protein CHORD regulates scaling of dendritic arbors with body size. *Sci Rep* 4: 1–8
